# Cytoplasmic CUG RNA Foci Are Insufficient to Elicit Key DM1 Features

**DOI:** 10.1371/journal.pone.0003968

**Published:** 2008-12-18

**Authors:** Warunee Dansithong, Cordula M. Wolf, Partha Sarkar, Sharan Paul, Andy Chiang, Ian Holt, Glenn E. Morris, Dorothy Branco, Megan C. Sherwood, Lucio Comai, Charles I. Berul, Sita Reddy

**Affiliations:** 1 Department of Biochemistry and Molecular Biology, Institute for Genetic Medicine, University of Southern California, Los Angeles, California, United States of America; 2 Children's Hospital Boston, Department of Cardiology and Department of Pediatrics, Harvard Medical School, Boston, Massachusetts, United States of America; 3 Department of Neurology, University of Texas Medical Branch, Galveston, Texas, United States of America; 4 Wolfson Centre for Inherited Neuromuscular Disease, Robert Jones and Agnes Hunt Orthopedic Hospital, Oswestry, United Kingdom; 5 Department of Microbiology and Immunology, Institute for Genetic Medicine, University of Southern California, Los Angeles, California, United States of America; Baylor College of Medicine, United States of America

## Abstract

The genetic basis of myotonic dystrophy type I (DM1) is the expansion of a CTG tract located in the 3′ untranslated region of *DMPK*. Expression of mutant RNAs encoding expanded CUG repeats plays a central role in the development of cardiac disease in DM1. Expanded CUG tracts form both nuclear and cytoplasmic aggregates, yet the relative significance of such aggregates in eliciting DM1 pathology is unclear. To test the pathophysiology of CUG repeat encoding RNAs, we developed and analyzed mice with cardiac-specific expression of a beta-galactosidase cassette in which a (CTG)_400_ repeat tract was positioned 3′ of the termination codon and 5′ of the bovine growth hormone polyadenylation signal. In these animals CUG aggregates form exclusively in the cytoplasm of cardiac cells. A key pathological consequence of expanded CUG repeat RNA expression in DM1 is aberrant RNA splicing. Abnormal splicing results from the functional inactivation of MBNL1, which is hypothesized to occur due to MBNL1 sequestration in CUG foci or from elevated levels of CUG-BP1. We therefore tested the ability of cytoplasmic CUG foci to elicit these changes. Aggregation of CUG RNAs within the cytoplasm results both in Mbnl1 sequestration and in approximately a two fold increase in both nuclear and cytoplasmic Cug-bp1 levels. Significantly, despite these changes RNA splice defects were not observed and functional analysis revealed only subtle cardiac dysfunction, characterized by conduction defects that primarily manifest under anesthesia. Using a human myoblast culture system we show that this transgene, when expressed at similar levels to a second transgene, which encodes expanded CTG tracts and facilitates both nuclear focus formation and aberrant splicing, does not elicit aberrant splicing. Thus the lack of toxicity of cytoplasmic CUG foci does not appear to be a consequence of low expression levels. Our results therefore demonstrate that the cellular location of CUG RNA aggregates is an important variable that influences toxicity and support the hypothesis that small molecules that increase the rate of transport of the mutant *DMPK* RNA from the nucleus into the cytoplasm may significantly improve DM1 pathology.

## Introduction

Myotonic dystrophy 1 (DM1) is a multi-system disorder characterized by skeletal myopathy and cardiac disease [1]. Sudden cardiac failure is one of the main causes of death in DM1 patients. Cardiac symptoms include variable conduction disorders and wall motion abnormalities [2]–[6]. First degree atrioventricular (AV) block and intraventricular conduction disorders are seen in ∼75% of DM1 patients [2]. Progressive deterioration of the conduction system resulting in complete AV block or ventricular arrhythmias are primarily responsible for sudden cardiac death [3], [4]. Although conduction disorders predominate in DM1, decreased ventricular systolic and diastolic functions, and hypertrophic and dilated cardiomyopathy, have been reported in severely affected patients [5]–[8]. Histological abnormalities include myofibrillar loss, fibrosis and fatty infiltration of both the working myocardium and the specialized conduction system. Electron microscopic examination shows aberrant Z lines and mitochondrial abnormalities in DM1 hearts [9].

The genetic defect in DM1 is the expansion of a CTG repeat tract on chromosome 19q13.3. The repeat expansion is located in the 3′ untranslated region of a protein kinase gene, *DMPK*, and is found 5′ of a homeodomain-encoding gene, *SIX5* [10]–[13]. CTG tract size is a strong predictor of cardiac involvement, particularly for electrocardiographic conduction, and wall motion abnormalities. Small expansions of 50–100 repeats produce a mild form of DM1 characterized primarily by the development of cataracts late in adult life. A multi-system adult onset form of the disease manifesting with cardiac disease occurs in a range of 250–500 repeats. Progressive increase in CTG tract size, to lengths greater than 1500 repeats, results in increased incidence and severity of the cardiac phenotype [4], [5]. Three non-exclusive molecular defects hypothesized to contribute to DM1 pathology are (i) decreased DMPK levels resulting from aberrant nuclear accumulation of the mutant *DMPK* RNA [14], [15] (ii) decreased SIX5 levels occurring as a consequence of chromatin condensation that occurs in the vicinity of the expanded CTG tract [16]–[18] and (iii) intrinsic toxicity of the expanded CUG tracts [19].

We have previously shown that both *Dmpk^+/−^* and *Dmpk^−/−^* mice demonstrate PR prolongation or first degree heart block, while *Dmpk^−/−^* mice exhibit a more severe phenotype consisting of both second and third degree heart block [20]–[22]. However, histological defects and wall motion abnormalities are not detected in these animals and the life-span of wild-type and mutant *Dmpk* mice is not significantly different. Structure-function analysis of *Six5^+/−^* cardiac muscle demonstrates that reduced *Six5* levels result in mild infra-Hisian conduction delay, increased left ventricular end diastolic dimension, and ventricular hypertrophy [23]. As reduction in *Dmpk* and *Six5* levels does not completely recapitulate the severity of DM1 cardiac pathology, these data suggest that toxic effects associated with the expression of CUG repeats play a prominent role in the etiology of DM1 heart disease. Consistent with a key role for expanded CUG repeat RNA in DM1 cardiac pathophysiology, inducible expression of high levels ∼960 interrupted CTG repeats located in the DMPK 3′UTR results in arrhythmias and cardiomyopathy that often lead to death of the transgenic animals within a few weeks after induction of the transgene [24]. In these experiments both elevated Cug-bp1 levels and aggregation of Mbnl1 in intra-nuclear RNA foci are documented in conjunction with aberrant splice site selection in a set of physiologically important RNAs [24]. Thus, as expression of expanded CUG repeats elicits key features of DM1 cardiac pathology, therapeutic strategies will require identification of mechanisms that will allow such RNAs to be rendered inert.

Here, we demonstrate that the cellular location of CUG RNA aggregates is a variable that influences the development and severity of DM1 cardiac pathology. In this study, we developed transgenic mice with cardiac-specific expression of a β-galactosidase cassette in which a (CTG)_400_ repeat tract is located 3′ of the termination codon of the β-galactosidase gene and 5′ of the bovine growth hormone poly A sequence. In these animals RNAs encoding expanded CUG repeats were found to aggregate exclusively within the cytoplasm of cardiomyocytes. Both in DM1 cells and in transgenic mice demonstrating cardiac specific expression of expanded CTG tracts located in the DMPK 3′UTR, aberrant RNA splicing is observed in conjunction with the aggregation of Mbnl1 in the nuclear CUG foci and increased steady-state levels of Cug-bp1 [24]–[26]. We therefore tested the ability of cytoplasmic CUG foci to elicit these changes. We observe both sequestration of Mbnl1 in the cytoplasmic CUG aggregates and approximately a two-fold elevation in the levels of Cug-bp1 in cardiomyocytes. Importantly, these defects did not result in abnormal RNA splicing and caused only a mild cardiac pathology, which manifests primarily as conduction defects under anesthesia. These results demonstrate that (i) CTG tracts expressed in a context independent manner can elicit elevated Cug-bp1 levels, (ii) a two fold elevation of Cug-bp1 levels is insufficient to dysregulate splice site choice in the adult mouse heart and (iii) aggregation of Mbnl1 in CUG foci per se may not be sufficient to inactivate Mbnl1.

The relative lack of toxicity of cytoplasmic CUG RNA aggregates is unlikely to be a consequence of low expression levels, as an independent set of experiments carried out in human myoblasts demonstrate that when this transgene is expressed at similar levels to a second transgene in which the expanded CTG tract was expressed in the context of the DMPK 3′UTR, it is unable to dysregulate RNA splicing. Consistent with our study, previous experiments in mouse myoblast cultures demonstrate that CTG tracts expressed in the context of the DMPK 3′UTR are no longer able to dysregulate myoblast differentiation when this cassette was re-engineered to encode the woodchuck post transcriptional element that served to localize the CUG encoding RNA within the cytoplasm [27]. These data therefore demonstrate that when expanded CUG tracts, expressed in a context independent manner or in the context of the DMPK 3′UTR, localize in the cytoplasm, they are unable to cause aberrant RNA splicing or significant pathology, when assessed in myoblast cultures or in the context of the adult mouse. Thus our data support the therapeutic use of small molecules that increase the transport of the mutant DMPK RNA from the nucleus into the cytoplasm as a means of greatly ameliorating DM1 pathology *in vivo*.

## Results

### DM1 myoblasts and fibroblasts contain both nuclear and cytoplasmic CUG foci

**Figure 1:**
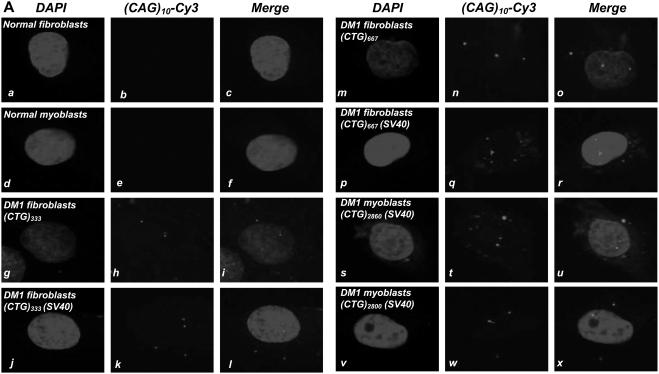
DM1 fibroblasts and myoblasts contain both nuclear and cytoplasmic CUG foci. Panel A: Nuclear DAPI staining of normal and DM1 fibroblasts and myoblasts, either untreated or immortalized with SV40, are shown in Panels *a*, *d*, *g*, *j*, *m*, *p*, *s & v*. *DMPK* transcripts encoding the expanded CUG tracts were detected by hybridization with a (CAG)_10_-Cy3 probe (red signal; Panels *h*, *k*, *n*, *q*, *t* & *w*). Transcripts containing expanded CUG repeats are not observed in the normal fibroblasts and normal myoblasts (*b & e*), respectively. Merged images of DAPI and (CAG)_10_-Cy3 stains show CUG RNA foci as red signals (*i*, *l*, *o*, *r*, *u & x*) within the nucleus and in the cytoplasm in DM1 fibroblasts and myoblasts. The percent of DM1 fibroblasts and myoblasts containing both nuclear and cytoplasmic foci are tabulated in Table 1. Images of CUG RNA foci in additional DM1 fibroblasts and myoblasts are shown in supplementary Figure S1 (Panels A–E).

Both primary cultures and SV40 transformed DM1 fibroblasts and myoblasts show CUG RNA foci in the nucleus and cytoplasm [Figure 1 and Supplementary Figure S1 (Panels A–E)]. SV40 transformation does not significantly alter the localization of CUG foci, as the percent of DM1 fibroblasts, which contain both nuclear and cytoplasmic foci, did not vary appreciably in untreated cultures and in SV40 immortalized lines [Figure 1; Table 1].

**Table 1:** DM1 fibroblasts and myoblasts contain both nuclear and cytoplasmic CUG foci.

Cell type	SV40 immortalized	Total cell #	# of cells containing nuclear foci	# of cells containing both nuclear and cytoplasmic foci
DM1 fibroblasts (CTG)_333_	−	97	65	32 (32.9%)
DM1 fibroblasts (CTG)_333_	+	68	45	23 (33.82%)
DM1 fibroblasts (CTG)_667_	−	134	93	41 (30.59%)
DM1 fibroblasts (CTG)_667_	+	77	53	24 (31.16%)
DM1 myoblasts (CTG)_2860_	+	196	125	71 (36.22%)
DM1 myoblasts (CTG)_2800_	+	86	57	29 (33.72%)

### Construction and analyses of α-MHC-LacZ-(CTG)_400_ mice

**Figure 2:**
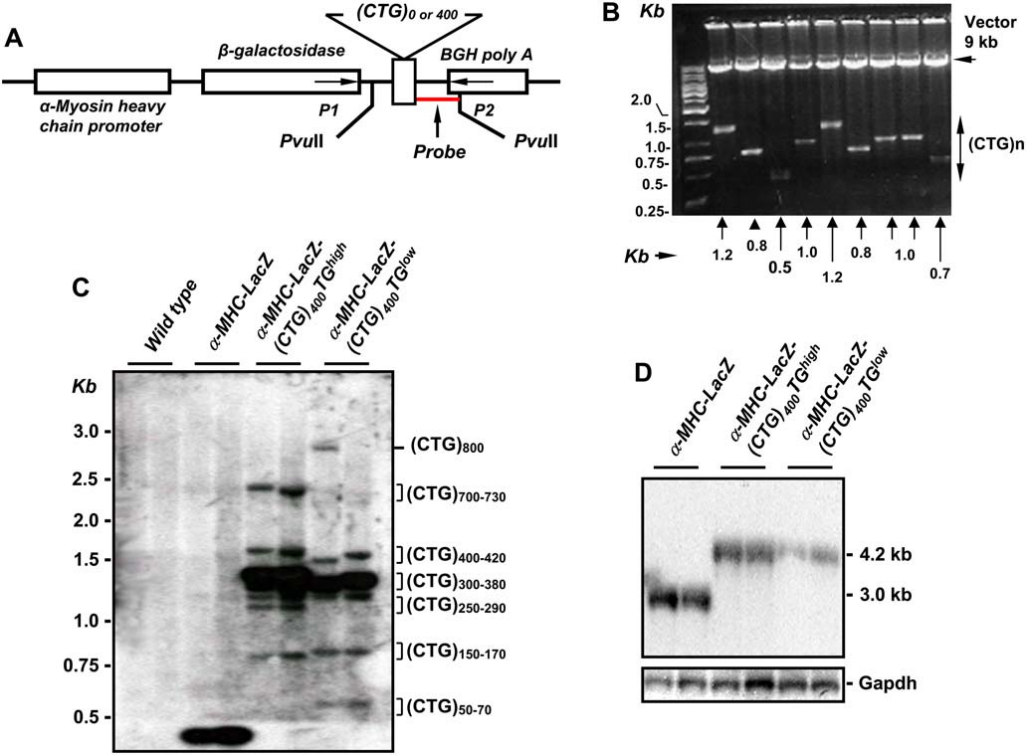
Characterization of α-MHC-LacZ-(CTG)_400_ mice. Panel A: The α-MHC-LacZ-(CTG)_400_ transgene encoding the α-myosin heavy chain promoter (α-MHC) used to drive cardiac specific expression of the *β-galactosidase* (*LacZ*) gene followed by a CTG tract of ∼400 repeats and the bovine growth hormone polyA (BGH-PolyA) sequence is shown. Panel B: Restriction digestion of plasmids encoding the α-MHC-LacZ-(CTG)_400_ sequences with *Sfi*I, which allows excision of the CTG repeat tract, demonstrates the instability of the CTG repeats when propagated in *E. coli* at 37°C. Panel C: Southern blot analysis of mouse tail-clip DNA digested with *Pvu*II. The 280 bp probe used for hybridization (*Panel A*) is shown. The majority of the detected bands contained 350∼380 CTG repeats in α-MHC-LacZ-(CTG)_400_TG*^high^* (band intensities ∼76%) or 300∼350 CTG repeats in α-MHC-LacZ-(CTG)_400_TG*^low^* (band intensities ∼80%) in tail clip DNAs. Panel D: Northern blot analysis of RNA derived from α-MHC-LacZ-(CTG)_400_ and α-MHC-LacZ mouse hearts probed with *β-galactosidase* and *Gapdh* sequences is shown.

To characterize the pathological effects intrinsic to the expression of expanded CTG repeat tracts in cardiac muscle, we built a transgenic cassette encoding the gene for β-galactosidase (*LacZ*) followed by a tract of ∼400 uninterrupted CTG repeats, which was cloned in a linker sequence and inserted between the *LacZ* termination codon and the bovine growth hormone polyadenylation (BGH-PolyA) sequence. A 5.5 kb α-myosin heavy chain (α-MHC) promoter, which encodes the first three untranslated exons of α-MHC, was used to drive specific expression of the LacZ-(CTG)_400_ cassette in the myocardium of embryonic atria and in adult mouse atria and ventricles [28, Figure 2; Panel A]. Consistent with the instability, which characterizes uninterrupted CTG tracts [29], [30], the (CTG)_400_ repeats tract showed a marked propensity to either delete or decrease in length when the plasmid encoding the transgenic cassette was propagated in bacteria at 37°C [Figure 2; Panel B]. The CTG tract was mutation-free when sequenced from each end to a distance of ∼300 bp, after which significant compressions were observed.

α-MHC-LacZ-(CTG)_400_ cassettes were injected into fertilized C57BL/6J mouse eggs to generate two independent lines. A α-MHC-LacZ cassette with no CTG tracts [α-MHC-LacZ-(CTG)_0_] was injected in parallel to develop control lines. Southern blot analyses of tail clip DNA from the progeny of both α-MHC-LacZ-(CTG)_400_ founders derived after approximately one year of breeding are shown in Figure 2; Panel C. Comparison of the two α-MHC-LacZ-(CTG)_400_ lines demonstrate that the lines showed deletions which ranged in length from ∼50–70 and ∼150–170 CTG repeats. However the majority of the CTG tracts contained ∼300–380 repeats with a significant fraction showing expansions that occurred primarily to lengths of ∼700–730 and ∼800 CTG repeats.

Consistent with the average CTG tract sizes observed in the transgenic mice, analysis of RNA isolated from hearts of α-MHC-LacZ mice and α-MHC-LacZ-(CTG)_400_ mice by Northern blots, showed transcript sizes of ∼3 and ∼4.2 kb when probed with β-galactosidase sequences [Figure 2; Panel D]. Progeny from the two founder α-MHC-LacZ-(CTG)_400_ lines, demonstrate different levels of LacZ-(CUG)_400_ RNA and are therefore denoted as α-MHC-LacZ-(CTG)_400_TG*^high^* and α-MHC-LacZ-(CTG)_400_TG*^low^* in the text.

### Mbnl1 sequesters in cytoplasmic CUG RNA foci in α-MHC-LacZ-(CTG)_400_ cardiomyocytes

**Figure 3:**
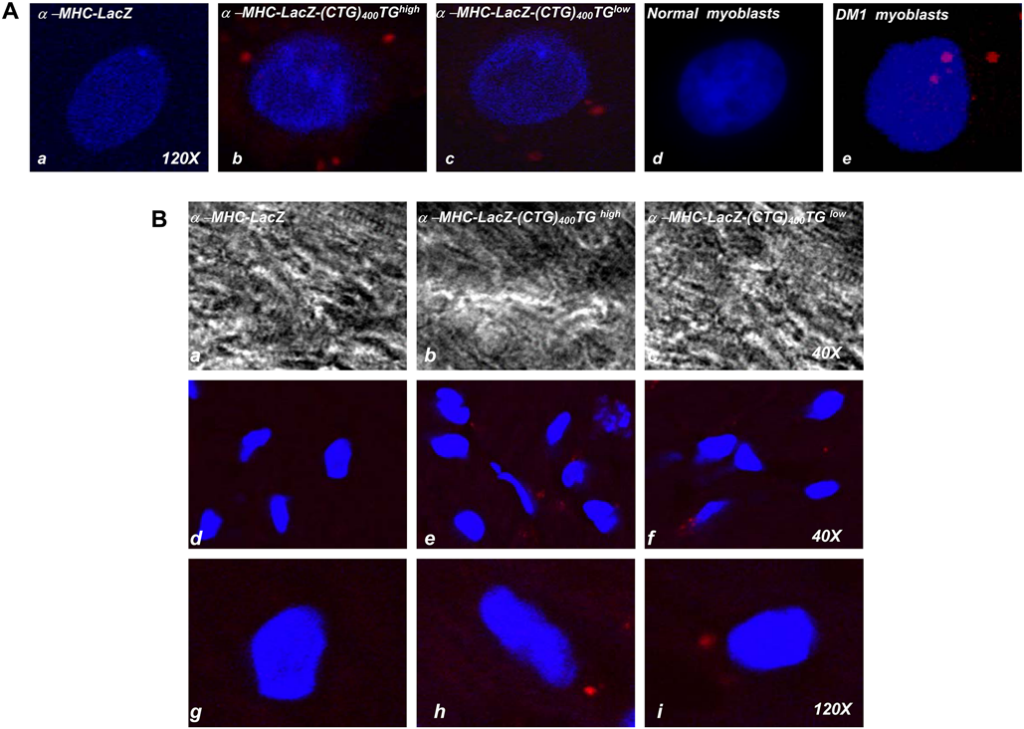
CUG foci form exclusively in the cytoplasm of α-MHC-LacZ-(CTG)_400_ cardiomyocytes. Panel A: Nuclear DAPI staining of cardiomyocytes derived from α-MHC-LacZ, α-MHC-LacZ-(CTG)_400_ mice and normal human and DM1 myoblasts is shown. Transcripts encoding the expanded CUG tracts were detected by hybridization with a (CAG)_10_-Cy3 probe (red signal). CUG foci are observed in the cytoplasm in α-MHC-LacZ-(CTG)_400_ cardiomyocytes (*b*, *c*) and in both the cytoplasm and nucleus of DM1 myoblasts (*e*) (120× magnification). Panel B: Nuclear DAPI staining of heart sections of α-MHC-LacZ, α-MHC-LacZ-(CTG)_400_TG*^high^*, and α-MHC-LacZ-(CTG)_400_TG*^low^* are shown. CUG foci are observed in the cytoplasm of both α-MHC-LacZ-(CTG)_400_TG*^high^*, and α-MHC-LacZ-(CTG)_400_TG*^low^* heart tissue sections [red signals; *e*, *f* (40× magnification) and *h*, *i* (120× magnification)]. Transcripts containing expanded repeats are not observed in cardiomyocytes and heart sections of α-MHC-LacZ mice and in normal human myoblasts [Panel A; *a*, *d* and Panel B; *d*, *g*]. >400 cells were examined in both α-MHC-LacZ-(CTG)_400_ cardiomyocyte preparations and in α-MHC-LacZ-(CTG)_400_ cardiac sections.

To examine the behavior of LacZ-(CUG)_400_ RNAs, we isolated cardiomyocytes from transgenic hearts and used fluorescence *in situ* hybridization (FISH) to visualize the location of the expanded CUG tracts with Cy3 labeled CAG probes [Figure 3]. CUG repeat RNAs aggregate exclusively within the cytoplasm and are excluded from the nucleus in α-MHC-LacZ-(CTG)_400_ cardiomyocyte preparations. Similarly, CUG RNA foci were also detected primarily in the cytoplasm when frozen tissue sections of in α-MHC-LacZ-(CTG)_400_ mice were examined [Figure 3; Panels A & B, >400 cells were examined in both α-MHC-LacZ-(CTG)_400_ cardiomyocyte preparations and in α-MHC-LacZ-(CTG)_400_ cardiac sections]. In these studies we observed that the cytoplasmic foci had diffuse borders when compared to the foci detected in DM1 cells.

**Figure 4:**
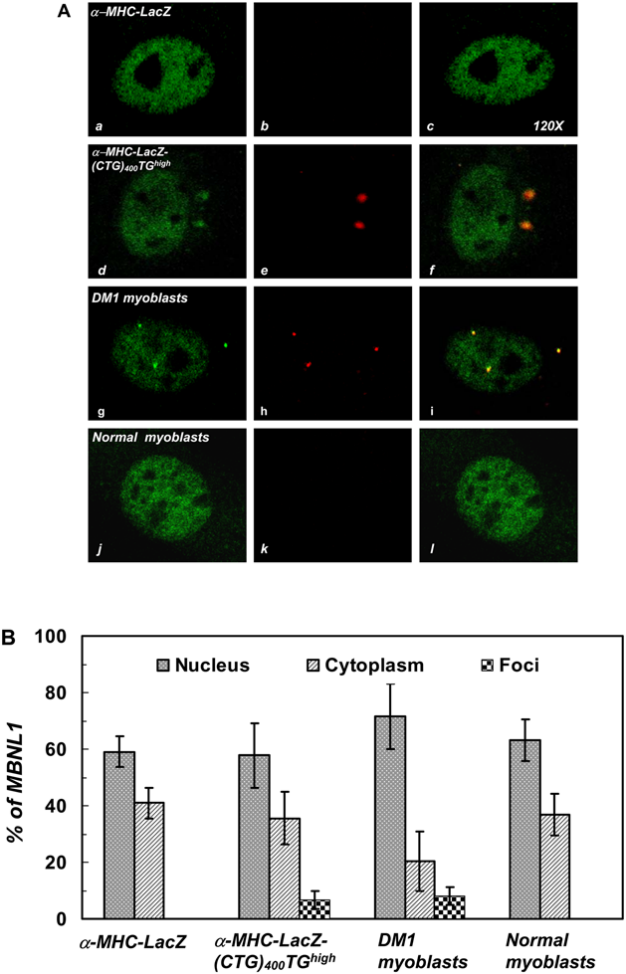
Mbnl1 co-localizes with cytoplasmic CUG-foci in α-MHC-LacZ-(CTG)_400_ cardiomyocytes. Panel A: Distribution of endogenous Mbnl1 is visualized as a green signal (*a*, *d*, *g & j*) using anti-MBNL1 (MB1a) monoclonal antibody and secondary antibodies conjugated with FITC. The mutant transcripts encoding the expanded CUG tracts were detected by hybridization with a (CAG)_10_-Cy3 probe (red signals; *e* & *h*). Transcripts containing expanded CUG repeats are not observed in the α-MHC-LacZ cardiomyocytes (*b*) and normal myoblasts (*k*). Merged images (*f* & *i*), where super-imposition of green and red signals are observed as a yellow signals, show that Mbnl1 co-localizes with the expanded CUG tracts in the α-MHC-LacZ-(CUG)_400_ cardiomyocytes (*f*) and in DM1 myoblasts (*i*). Panel B: Graphical representation of Mbnl1 distribution in each compartment (nucleus, cytoplasm and foci) of α-MHC-LacZ, α-MHC-LacZ-(CUG)_400_ cardiomyocytes and DM1 and normal myoblasts is shown and the results are tabulated in Table 2. No significant difference is observed in the fraction of Mbnl1 which colocalizes with the foci in α-MHC-LacZ-(CTG)_400_ cardiomyocytes and in DM1 myoblasts (p = 0.37). The specificity of MBNL1 (MB1a) monoclonal antibody was assessed by immunofluorescence using cardiomyocytes derived from *Mbnl1^−/−^* mice (Supplementary Figure S2).

To test if CUG-RNA aggregates aberrantly sequester the alternative splice factor, Mbnl1, we stained both α-MHC-LacZ and α-MHC-LacZ-(CTG)_400_ cardiomyocytes and normal human and DM1 myoblasts with anti-MBNL1 (MB1a) monoclonal antibodies [31]. Co-localization studies demonstrate that Mbnl1 aberrantly sequesters within the cytoplasmic CUG RNA foci in α-MHC-LacZ-(CTG)_400_ cardiomyocytes [Figure 4; Panel A]. Quantitation of the sequestered Mbnl1 demonstrates that the percent of Mbnl1 sequestered in the cytoplasmic foci in α-MHC-LacZ-(CTG)_400_ cardiomyocytes is not significantly different (6.65%) from the percent of MBNL1 sequestered within the nuclear and cytoplasmic foci in DM1 myoblasts (8.01%) [p = 0.37, Figure 4; Panel B & Table 2]. The specificity of MBNL1 (MB1a) monoclonal antibody was verified by immunofluorescence using cardiomyocytes derived from *Mbnl1^−/−^* mice [32, Supplementary Figure S2]. Thus these results demonstrate that cytoplasmic CUG foci, although more diffuse in appearance, can effectively sequester Mbnl1 *in vivo*.

**Table 2:** Distribution of MBNL1 in α-MHC-LacZ-(CTG)_400_ cardiomyocytes and DM1 myoblasts.

Cell type	Cell #	*% MBNL1*			*p*-value (Foci)
		Nuclus	Cytoplasm	Foci	
α-MHC-LacZ cardiomyocytes	8	59.02±5.46	40.97±5.46	0.0	
α-MHC-LacZ-(CTG)_400_TG*^high^* cardiomyocytes	20	57.81±11.35	35.53±9.32	6.65±3.06#	
DM1 myoblasts	18	71.54±11.45	20.43±10.49	8.01±3.19#	0.37
Normal myoblasts	16	63.14±7.43	36.86±7.43	0.0	

(#) represents pair wise comparison; p-value (Student's t-test) denotes no significant difference in MBNL1 sequestered in foci of α-MHC-LacZ-(CTG)_400_TG^high^ and DM1 myoblasts.

### Cug-bp1 levels are elevated in α-MHC-LacZ-(CTG)_400_ cardiomyocytes

**Figure 5:**
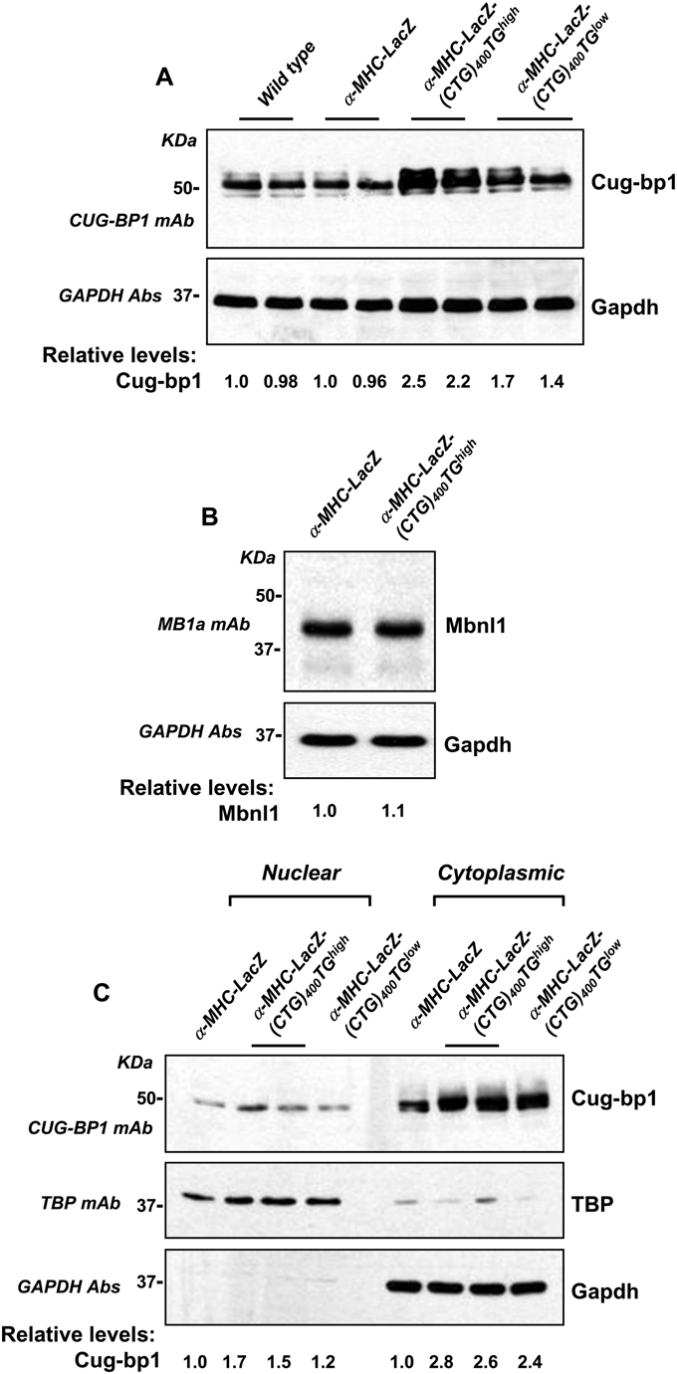
α-MHC-LacZ-(CTG)_400_ mice show increased steady-state levels of Cug-bp1. Panels A–B: Protein extracts were prepared from α-MHC-LacZ, α-MHC-LacZ-(CTG)_400_TG*^high^*, and α-MHC-LacZ-(CTG)_400_TG*^low^* mouse hearts and 6 or 10 µg of the total proteins from the tissue extracts were resolved on SDS-PAGE followed by Western blot analyses and immunostaining with CUG-BP1 and MBNL1 monoclonal antibodies (mAb), respectively. The blots were re-probed for GAPDH using anti-GAPDH polyclonal antibodies as an internal control. The experiments were carried out in triplicate and mean values of steady-state Cug-bp1 and Mbnl1 levels are shown. Panel C: Cytoplasmic and nuclear proteins extracts (10 µg) from α-MHC-LacZ, α-MHC-LacZ-(CTG)_400_TG*^high^*, and α-MHC-LacZ-(CTG)_400_TG*^low^* mouse hearts were resolved on SDS-PAGE followed by Western blot analyses and immunostaining with CUG-BP1 mAb. The blots were re-probed for TATA binding protein (TBP), and for GAPDH, which were used as nuclear and cytoplasmic markers respectively. The experiments were carried out in triplicate and mean values of steady-state Cug-bp1 levels are shown.

To test if expression of LacZ-(CUG)_400_ RNAs can elicit a change in steady-state Cug-bp1 levels, we measured the steady-state levels of Cug-bp1 in tissue lysates derived from hearts of 6 months old α-MHC-LacZ-(CTG)_400_TG*^high^*, α-MHC-LacZ-(CTG)_400_TG*^low^* and α-MHC-LacZ mice. Three independent western blot analyses in which hearts from two mice of each genotype were analyzed demonstrate a ∼2.5 fold increase in Cug-bp1 levels in α-MHC-LacZ-(CTG)_400_TG*^high^* and ∼1.6 fold increase in α-MHC-LacZ-(CTG)_400_TG*^low^* mice. The fold changes in steady-state Cug-bp1 levels were not significantly different when either 6 µg or 10 µg of proteins were sampled [Figure 5; Panel A]. In these experiments no significant alteration in steady-state Mbnl1 levels were detected in α-MHC-LacZ and α-MHC-LacZ-(CTG)_400_TG*^high^* mice [Figure 5; Panel B]. To test if nuclear Cug-bp1 levels were altered in α-MHC-LacZ-(CTG)_400_ mice, we measured Cug-bp1 protein levels in the cytoplasmic and nuclear fractions of heart tissue derived from α-MHC-LacZ, α-MHC-LacZ-(CTG)_400_TG*^high^*, and α-MHC-LacZ-(CTG)_400_TG*^low^* animals. In these experiments Cug-bp1 was found to localize primarily in the cytoplasm in both α-MHC-LacZ and α-MHC-LacZ-(CTG)_400_ mice. Cug-bp1 levels were elevated ∼2.7 and ∼2.4 fold , and ∼1.6 and ∼1.2 fold in the cytoplasm and in the nucleus of MHC-LacZ-(CTG)_400_TG*^high^* and α-MHC-LacZ-(CTG)_400_TG*^low^* mice when compared to controls [Figure 5; Panel C].

### RNA splicing is not dysregulated in α-MHC-LacZ-(CTG)_400_ hearts

**Figure 6:**
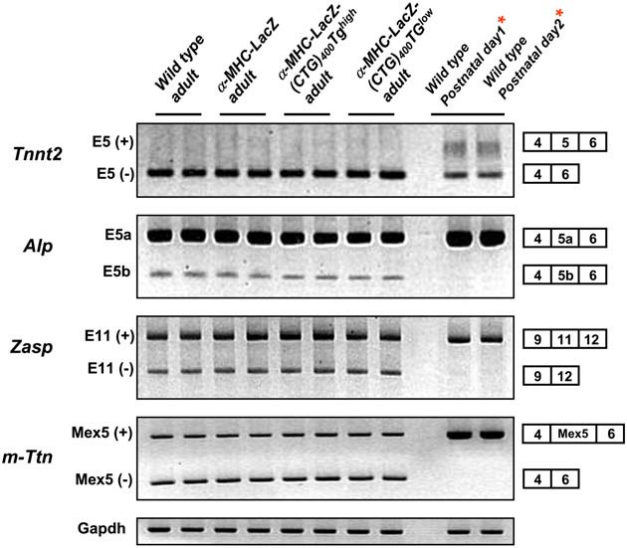
Aberrant splicing is not observed in α-MHC-LacZ-(CTG)_400_ hearts. Total RNA isolated from adult α-MHC-LacZ-(CTG)_400_ and adult α-MHC-LacZ hearts and wild-type postnatal day1 and day 2 mouse hearts was subjected to RT-PCR analysis using the *Tnnt2*, *Alp*, *Zasp* and *m-Ttn* primers as described in Methods. *Gapdh* RNA was amplified in parallel as an internal control. The experiments were carried out in triplicate and the results are tabulated in Table 3.

As the cytoplasmic LacZ-CUG foci aggregate Mbnl1 and elicit elevated steady-state levels of Cug-bp1, we studied the effect of LacZ-(CUG)_400_ RNA expression on RNA splice site choice. We examined the pattern of alternative splicing of cardiac troponin T (*Tnnt2*), Z-band alternatively spliced PDZ-motif protein (*Zasp*), alpha-actinine-2 associated LIM protein (*Alp*) and M-line Titin (*m-Ttn*) [33]-[35] RNAs. These RNAs are aberrantly spliced in adult DM1 hearts and retain the pattern of splicing observed in new-borns [36]. No significant change in the splicing pattern of these RNAs is observed in heart tissue of either the α-MHC-LacZ-(CTG)_400_TG*^high^* or α-MHC-LacZ-(CTG)_400_TG*^low^* mice when compared to control α-MHC-LacZ mice. In all cases, the relative percentages of the detected isoforms for each gene did not vary significantly for each of the two amplification conditions tested [Figure 6 & Table 3].

**Table 3:** Alternative RNA splicing in α-MHC-LacZ-(CTG)_400_ hearts.

Gene	Accession no.	Alt. spliced exon number	n	*% Exons inclusion*					
				WT adult	LacZ adult	TG*^high^* adult	Tg*^low^* adult	WT Posnatal day 1	WT Posnatal day 2
*Tnnt2*	NM_011619	5	3	6.8±1.15	6.15±1.1	6.9±1.3	7.5±0.9	24.3±1.7*	22.2±1.6*
*Alp*	AF002283	5a	3	81.7±2.0	80.2±1.6	80.5±1.8	81.1±2.5	98.2±0.5*	97.7±1.0*
*Zasp*	AY206013	11	3	67.9±4.7	65.5±3.1	67.6±4.1	69.1±5.3	91.6±0.7*	89.7±1.3*
*m-Ttn*	XM_130312	Mex5	3	48.7±1.2	47.6±1.3	48.2±1.3	47.4±0.9	98.6±0.4*	98.4±0.5*

Asterisk (^*^) represents significant differences from wild type (WT) adult (Student's t-test; p<0.05). Tnnt2, cardiac troponin T; Alp, alpha-actinine-2 associated LIM protein; Zasp, Z-band alternatively spliced PDZ-motif protein; m-Ttn, M-line Titin; LacZ, α-MHC-LacZ-(CTG)_0_; TG^high^, α-MHC-LacZ-(CTG)_400_TG^high^; TG^low^, α-MHC-LacZ-(CTG)_400_TG^low^.

### Expression of LacZ-(CUG)_400_ RNAs at levels equivalent to that of a DMPK mini gene encoding 300 CTG repeats does not result in aberrant RNA splicing in human myoblasts

**Figure 7:**
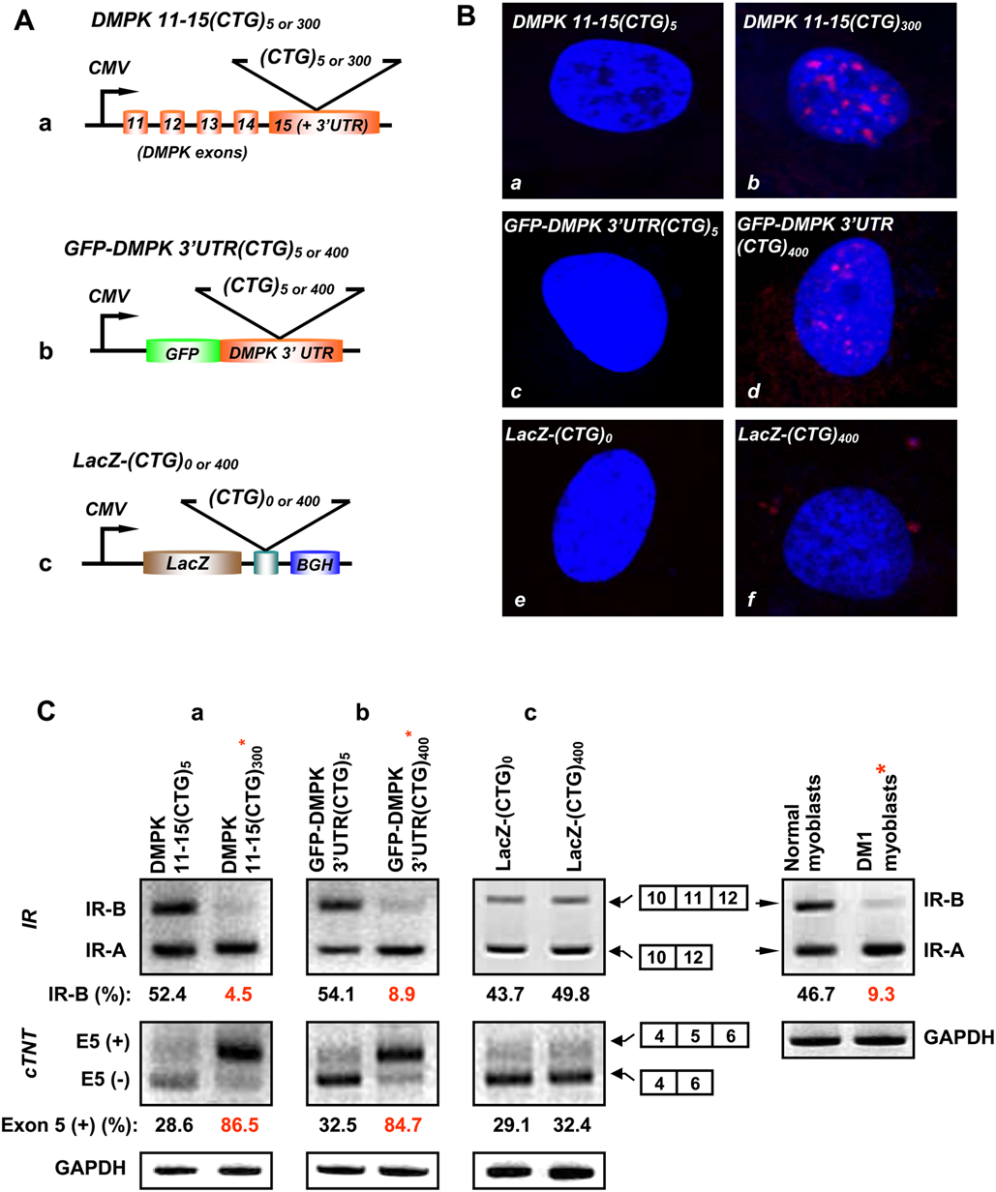
Expression of LacZ-(CUG)_400_ RNAs is insufficient to dysregulate IR and cTNT splicing in human myoblasts. Panel A: DMPK 11-15(CTG)_5 or 300_ (a), GFP-DMPK 3′UTR (CTG)_5 or 400_ (b) and LacZ-(CTG)_0 or 400_ (c) cassettes under the transcriptional control of the cytomegalovirus (CMV) promoter are shown. Panel B: Nuclear DAPI staining of human normal myoblasts expressing DMPK11-15(CTG)_5_ (*a*), DMPK 11-15(CTG)_300_ (*b*), GFP-DMPK 3′UTR(CTG)_5_ (*c*), GFP-DMPK 3′UTR(CTG)_400_ (*d*), LacZ-(CTG)_0_ (*e*), LacZ-(CTG)_400_ (*f*) cassettes are shown. The mutant transcripts encoding the expanded CUG tracts were detected by hybridization with a (CAG)_10_-Cy3 probe. CUG RNA foci are observed primarily within the nucleus in normal myoblasts expressing DMPK11-15(CTG)_300_ (red signal; *b*) and GFP-DMPK 3′UTR (CTG)_400_ (red signal; *d*). CUG RNA foci are observed in the cytoplasm (red signal; *f*) in normal myoblasts expressing the LacZ-(CTG)_400_ cassette. Normal myoblasts expressing DMPK11-15(CTG)_5_ (*a*), GFP-DMPK 3′UTR(CTG)_5_ (*c*), and LacZ-(CTG)_0_ (*e*) constructs did not show RNA foci. Panel C: *IR* and *cTNT* RNA splicing in myoblasts expressing the indicated cassettes are shown. Synthesized cDNAs (150 ng) were subjected to RT-PCR analysis using the *IR* and *cTNT* primers described in Methods. *GAPDH* RNA was amplified in parallel as an internal control. The experiments were carried out in triplicate. Representative panels are shown in Panel C and the results are tabulated in Table 4.

**Figure 8:**
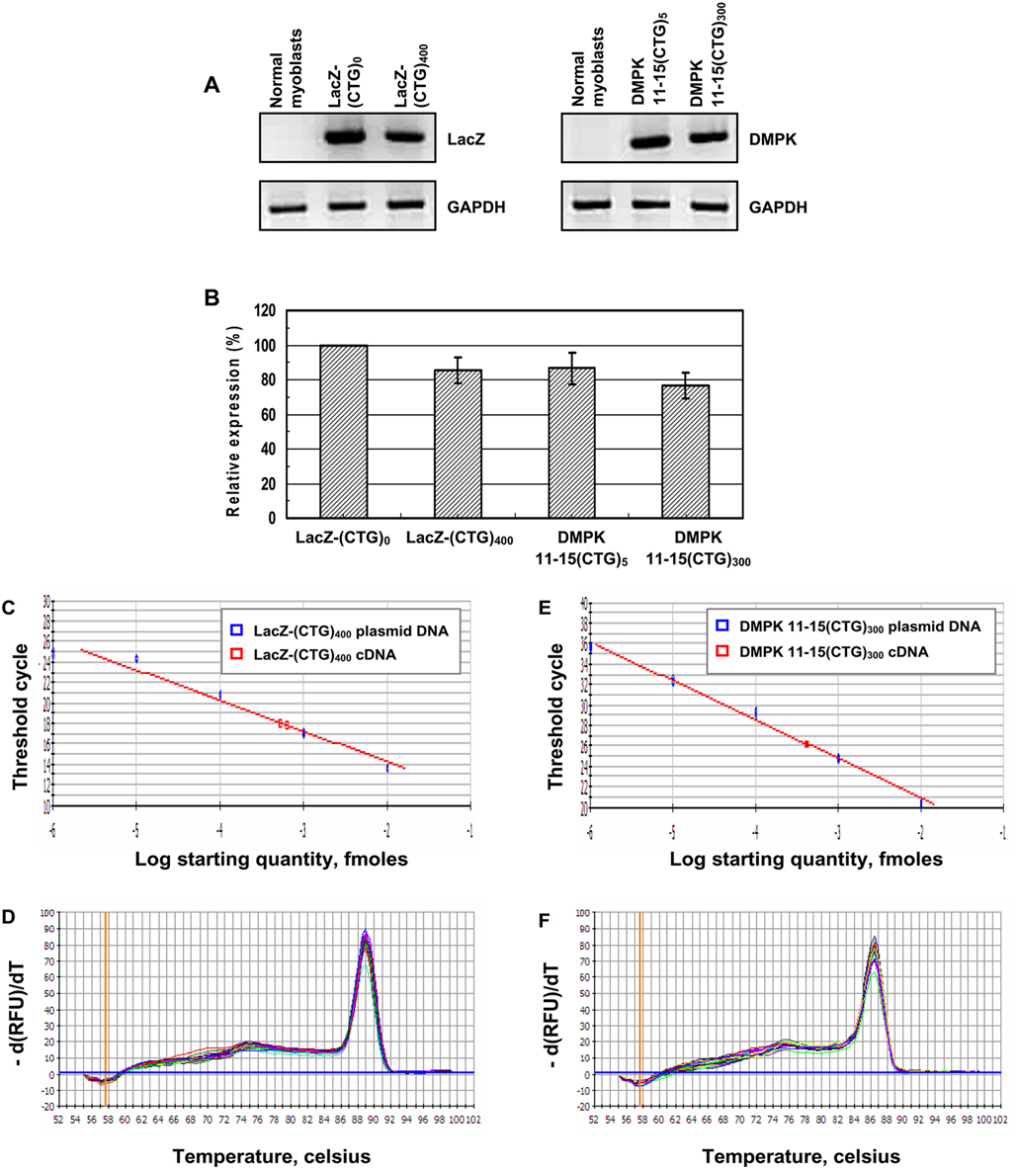
Quantitation of the steady-state levels of LacZ-(CUG)_400_ and DMPK 11-15(CUG)_300_ RNAs. Panels A–B: RT-PCR analyses of the steady-state expression levels of LacZ-(CTG)_0_, LacZ-(CTG)_400_, and DMPK11-15(CTG)_5_, DMPK 11-15(CTG)_300_ cassettes are shown. Synthesized cDNA (100 ng) from normal myoblsts expressing LacZ-(CTG)_0 or 400_ and DMPK 11-15(CTG)_5 or 300_ were subjected to RT-PCR analysis. *GAPDH* RNA was amplified in parallel as an internal control. The experiments were carried out in triplicate and the results are tabulated in Table 5. Relative steady-state expression levels of the LacZ-(CTG)_400_ and DMPK 11-15(CTG)_300_ cassettes were not significantly different (p = 0.479). Panels C–F: Real-time PCR analysis of serial dilutions of plasmid DNAs encoding LacZ-(CTG)_400_ and DMPK 11-15(CTG)_300_ sequences and of LacZ-(CTG)_400_ and DMPK 11-15(CTG)_300_ cDNAs is shown. PCR reactions were carried using 10^−2^ to 10^−6^ fmoles of plasmid DNAs encoding LacZ-(CTG)_400_ or DMPK 11-15(CTG)_300_ sequences. To quantitate the expression levels of expanded CUG repeat encoding transcripts, cDNAs (5 ng) from human myoblasts expressing LacZ-(CTG)_400_ and DMPK 11-15(CTG)_300_ were subjected to Real-time PCR analysis in parallel. LacZ-(CTG)_400_ and DMPK 11-15(CTG)_300_ cDNAs are present at approximately similar levels (Panels C, E & Table 6). Melting curves of LacZ-(CTG)_400_ or DMPK 11-15(CTG)_300_ PCR reactions are shown (Panels D & F). *GAPDH* was used as an internal control for RNA quality and the reverse transcriptase reaction (Ct values for GAPDH in LacZ-(CTG)_400_ and DMPK 11-15(CTG)_300_ samples was 19.8 in each case).

To examine if low expression levels are responsible for the lack of toxicity of LacZ-(CUG)_400_ RNAs in α-MHC-LacZ-(CTG)_400_ mice, we studied a set of expression vectors in human myoblast cultures in which the cytomegalovirus (CMV) promoter was used to express cassettes encoding a DMPK minigene encoding either 5 or 300 interrupted CTG repeats [DMPK 11-15(CTG)_5 or 300_], the green fluorescent protein linked to the DMPK 3′UTR containing either 5 or 400 CTG repeats [GFP-DMPK 3′UTR(CTG)_5 or 400_], or the β-galactosidase gene containing no repeats or 400 repeats [LacZ-(CTG)_0 or 400_] [Figure 7; Panel A]. Expression of constructs encoding both the DMPK 11-15 minigene and GFP-DMPK 3′UTR with the expanded CTG repeats resulted in nuclear foci and aberrant RNA splicing. All constructs that did not contain the expanded CTG tracts did not dysregulate RNA splicing. Expression of the LacZ-(CTG)_400_ construct formed cytoplasmic CUG foci but did not alter splice site selection in *IR* and *cTNT* RNAs [Figure 7; Panels B–C and Table 4]. RT-PCR analyses of the steady-state expression levels of the β-galactosidase cassette encoding 400 CTG tracts and the DMPK minigene encoding 300 CTG repeats, which are approximately 4.3 and 2.2 kb in length respectively, demonstrate that the expression levels achieved by these cassettes was not significantly different [p = 0.479, Figure 8; Panels A–B and Table 5]. To further confirm that comparable expression levels of LacZ-(CUG)_400_, and DMPK 11-15(CUG)_300_ RNAs were assayed in these experiments, we quantitated the amounts of LacZ-(CTG)_400_, and DMPK 11-15(CTG)_300_ cDNAs by Real-time PCR analyses. Use of a standard curve generated from plasmid DNAs encoding these sequences demonstrates that the steady-state expression levels of the LacZ-(CTG)_400_, and DMPK 11-15(CTG)_300_ cDNAs are similar [LacZ-(CTG)_400_ = 5.68×10^−4^ fmoles and DMPK 11-15(CTG)_300_ = 4.17×10^−4^ fmoles; Figure 8; Panels C–F and Table 6]. Thus when expressed at a comparable level to the DMPK minigene encoding 300 CTG repeats [DMPK 11-15(CUG)_300_], the LacZ-(CUG)_400_ RNAs are unable to dysregulate splicing in human myoblasts.

**Table 4:** Aberrant RNA splicing results from the expression of expanded CTG repeats in myoblasts.

Gene	Alt. spliced exon number	n	DMPK 11-15(CTG)_5_	DMPK 11-15(CTG)_300_	*% Exons splicing*				Normal myoblasts	DM1 myoblasts
					GFP-DMPK 3′UTR(CTG)_5_	GFP-DMPK 3′UTR(CTG)_400_	LacZ-(CTG)_0_	LacZ-(CTG)_400_		
*IR*	11	3	52.4±2.3	6.2±1.7*	51.4±2.7	11.2±2.3*	43.7±3.5	46.9±2.9	46.7±2.7	6.2±3.1*
*cTNT*	5	3	30.5±1.9	83.9±2.6*	32.5±2.9	81.6±3.1*	27.3±1.8	30.1±2.3	ND	ND

Asterisk (^*^) represents significant differences from the control (Student's t-test; p<0.05). IR, insulin receptor; cTNT, cardiac troponin T; ND, Not determined.

**Table 5:** Quantitation of levels of LacZ-(CUG)_400_ and DMPK 11-15(CUG)_300_ RNAs by RT-PCR.

Plasmid constructs transfected into myoblasts	Relative expression (%)	*p*-value
LacZ-(CTG)_0_	100.0	
LacZ-(CTG)_400_	85.3±7.4#	
DMPK 11-15(CTG)_5_	86.5±9.3	
DMPK 11-15(CTG)_300_	76.4±7.5#	0.479

(#) represents pair wise comparison; p-value (Student's t-test) denotes no significant difference in the expression levels of LacZ-(CTG)_400_ and DMPK 11-15(CTG)_300_ cDNAs.

**Table 6:** Quantitation of LacZ-(CUG)_400_ and DMPK 11-15(CUG)_300_ RNAs by Real-time PCR.

cDNA	fmole	*p*-value
LacZ-(CTG)_400_	5.68×10^−4^	
DMPK 11-15(CTG)_300_	4.17×10^−4^	0.06

p-value (Student's t-test; p<0.05) denotes no significant difference in the levels of LacZ-(CTG)_400_ and DMPK 11-15(CTG)_300_ cDNAs.

### Electrocardiography demonstrates intraventricular conduction defects in sedated α-MHC-LacZ-(CTG)_400_ mice

To test if cytoplasmic LacZ-CUG aggregates cause functional defects in the heart, we carried out a series of *in vivo* experiments. Surface 6-lead ECG and ambulatory telemetric ECG recordings were performed in 14 α-MHC-LacZ-(CTG)_400_TG*^high^*, 9 α-MHC-LacZ-(CTG)_400_TG*^low^* and 5 α-MHC-LacZ mice. PR intervals are not prolonged in α-MHC-LacZ-(CTG)_400_TG*^high^* and α-MHC-LacZ-(CTG)_400_TG*^low^* when compared to α-MHC-LacZ mice. P wave amplitude and duration are quantitatively similar between groups (data not shown). ECG data from sedated animals however demonstrated prolonged QRS intervals in α-MHC-LacZ-(CTG)_400_TG*^low^* compared to α-MHC-LacZ-(CTG)_400_TG*^high^* mice and longer QT/QTc durations in α-MHC-LacZ-(CTG)_400_TG*^low^* compared to α-MHC-LacZ-(CTG)_400_TG*^high^* and α-MHC-LacZ mice. Intraventricular conduction delay or a bundle branch block pattern was noticed in 6 of 9 α-MHC-LacZ-(CTG)_400_TG*^low^*, 6 of 14 α-MHC-LacZ-(CTG)_400_TG*^high^* mice, compared with 0 of 5 α-MHC-LacZ mice (p = 0.054, Pearson Chi-Square). The ECG measurements and calculations for all animals studied are summarized in Tables 7 and 8.

**Table 7:** Telemetry measurements in conscious α-MHC-LacZ-(CTG)_400_ mice.

	LacZ (N = 5)	TG*^high^* (N = 14)	TG*^low^* (N = 9)	One-way ANOVA
SCL (ms)	99±5	88±9	88±8	*ns* (*0.06*)
HR (bpm)	609±31	685±71	686±59	*ns* (*0.06*)
PR (ms)	35.4±1.8*	31.9±2.1*	33.4±2.3	*p* = *0.011*
QRS (ms)	14.6±0.7	14.0±1.5	14.8±1.0	*ns*
QT (ms)	24.8±1.5	23.4±2.3	25.2±2.9	*ns*
QTc (ms)	25.0±1.2	25.0±2.7	26.8±2.7	*ns*

Parameter values represent mean±standard deviation. Matching symbols (^*^) denote significant differences between groups in post hoc testing for that parameter; LacZ, α-MHC-LacZ-(CTG)_0_; TG^high^, α-MHC-LacZ-(CTG)_400_TG^high^; TG^low^, α-MHC-LacZ-(CTG)_400_TG^low^; SCL, sinus-cycle length; HR, heart rate; PR, atrial and A–V nodal conduction time; QRS, ventricular depolarization time; QT, surrogate of action potential duration; QTc, corrected surrogate of action potential duration; ms, milliseconds, bpm, beats per minute.

**Table 8:** ECG measurements in sedated α-MHC-LacZ-(CTG)_400_ mice.

	LacZ (N = 5)	TG*^high^* (N = 14)	TG*^low^* (N = 9)	One-way ANOVA
SCL (ms)	147±3	143±19	142±7	*ns*
HR (bpm)	407±8	427±56	424±20	*ns*
PR (ms)	39.6±2.8	37.3±3.4	41.2±5.5	*ns*
QRS (ms)	13.9±0.8	13.7±2.5*	16.8±1.8*	*P* = *0.005*
QT (ms)	24.5±1.2#	22.6±3.7*	30.0±2.9*#	*P*≤*0.001*
QTc (ms)	20.2±0.9#	19.1±4.0*	25.2±2.5*#	*P*≤*0.001*

Parameter values represent mean±standard deviation; Matching symbols (^*^,#) denote significant differences between groups in post hoc testing for that parameter; LacZ, α-MHC-LacZ-(CTG)_0_; TG^high^, α-MHC-LacZ-(CTG)_400_TG^high^; TG^low^, α-MHC-LacZ-(CTG)_400_TG^low^; SCL, sinus-cycle length; HR, heart rate; PR, atrial and A–V nodal conduction time; QRS, ventricular depolarization time; QT, surrogate of action potential duration; QTc, corrected surrogate of action potential duration; ms, milliseconds, bpm, beats per minute.”

### Exercise Tolerance Testing did not elicit cardiac arrhythmia

Exercise tolerance testing was accomplished in 14 of α-MHC-LacZ-(CTG)_400_TG*^high^*, 9 of α-MHC-LacZ-(CTG)_400_TG*^low^* and 5 of α-MHC-LacZ mice. All 28 mice successfully completed 30 minutes of running. The PR intervals did not change significantly during exercise testing, and no higher AV block or any arrhythmias were provoked with exertion.

### Mitochondrial defects are observed α-MHC-LacZ-(CTG)_400_ heart tissue

**Figure 9:**
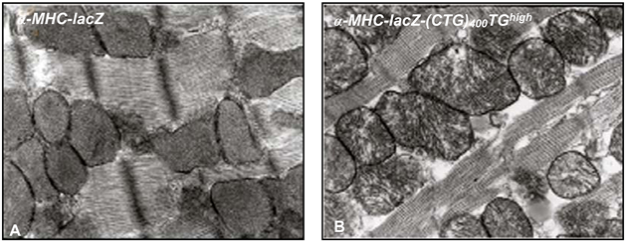
Aberrant mitochondrial cristae are observed in α-MHC-LacZ-(CTG)_400_ hearts. Electron micrographs of adult heart sections of α-MHC-LacZ (A) and α-MHC-LacZ-(CTG)_400_TG*^high^* (B) mice are shown. Mitochondrial cristae were disarrayed in a subset of sections viewed.

No gross abnormalities in the cardiac muscle structure were observed in H & E sections of α-MHC-LacZ-(CTG)_400_ mice (data not shown). Electron microscopy showed disarrayed cristae in mitochondria in some sections of both adult α-MHC-LacZ-(CTG)_400_TG*^high^* and α-MHC-LacZ-(CTG)_400_TG*^low^* mice but not in α-MHC-LacZ mice [Figure 9]. Thus consistent with the lack of RNA splice defects in α-MHC-LacZ-(CTG)_400_ mice, these results demonstrate that LacZ-(CUG)_400_ RNAs are unable to elicit significant cardiac pathology *in vivo*.

## Discussion

Myotonic dystrophy is a multi-system disorder, characterized by aberrant RNA splicing, which results from the expansion of a CTG tract located in the 3′UTR of *DMPK*. An important mediator of DM1 pathology is the mutant *DMPK* RNA encoding the expanded CUG tracts [24], [37]–[39]. Thus a central aspect of designing therapeutic interventions for this disease is to determine how to alter such toxic RNAs into benign or relatively inert macromolecules. RNAs encoding expanded CUG repeats form both nuclear and cytoplasmic aggregates or foci in DM1 cells. The relative toxicity of such aggregates both in terms of evoking aberrant RNA splicing and in eliciting DM1 pathophysiology *in vivo* is currently unknown. In this study we describe the behavior of expanded CTG tracts expressed in the context of the β-galactosidase gene under the control of the α-myosin heavy chain promoter in mouse hearts. LacZ-(CUG)_400_ RNAs form aggregates exclusively in the cytoplasm of cardiomyocytes in transgenic mice. Significantly, the cytoplasmic LacZ-CUG RNA aggregates are unable to dysregulate splice site choice in mouse hearts and result only in mild cardiac dysfunction. Our results therefore support a therapeutic strategy aimed at the identification of small molecules that facilitate effective and rapid transport of toxic CUG RNAs from the nucleus into the cytoplasm as a means of markedly reducing the toxicity of such RNAs.

Several lines of evidence demonstrate that mutant RNAs encoding expanded CUG repeat tracts embedded in the DMPK 3′UTR, which aggregate within the nucleus, facilitate the development of DM1 pathology. Seznec and colleagues have shown that expression of expanded CTG tracts in the context of the human DMPK gene results both in nuclear foci and the development of DM1 pathology in mice [38]. In this study, the severity of the phenotype was influenced both by tract size and expression levels. Specifically, 300 CUG repeats were found to be the minimum repeat tract length at which an overt pathology was detected in mice. Transgene expression levels were a second variable in this study, as homozygous animals were more severely affected than hemizygous mice. Nonetheless, not all of the animals that expressed CUG foci in the nucleus demonstrate a DM1 phenotype. Such differences were attributed by the authors to possible variations in the pattern of transgene expression during development or differences in RNA stability [38]. Consistent with these earlier results, inducible expression of high levels of interrupted CUG repeat tracts expressed in the context of the DMPK 3′UTR, under the control of a strong ubiquitous promoter, has also been shown to result in the rapid aggregation of CUG repeats within the nucleus and the development of severe DM1 pathology in mice [24], [39]. CUG expansions that are expressed in a context other than the DMPK 3′UTR show more variable results. Expression of high levels of RNAs encoding ∼250 CUG tracts located in the 3′UTR of the human skeletal actin gene cause both intra-nuclear foci and DM1 pathophysiology in mouse skeletal muscles [37]. In contrast, expression of ∼162 CUG repeats embedded in sequences that contain ∼100 bps of DMPK 3′UTR sequences in flies showed nuclear foci but no pathology [40]. In a second study in flies, 480 interrupted CUG tracts expressed as a non-coding transcript allowed both the development of nuclear foci and pathology in several tissues [41]. In a third study, inducible expression of interrupted CUG tracts, varying in length from 16 to 480 repeats, was studied in various transgene insertion contexts in flies. In this set of experiments several fly strains showed nuclear foci but only one strain of transgenic flies showed significant pathology [42]. It is currently unclear why such variability is observed in flies; however CTG tract sizes, expression levels and context of expression may all contribute to the differences in the observed phenotypes. These data demonstrate that although nuclear foci are not sufficient to produce a DM1 phenotype, in all cases in which DM1 pathology develops nuclear foci are observed. Thus taken together these data support the hypothesis that nuclear foci are required for DM1 pathology to manifest, but may not under some circumstances be sufficient to produce an overt phenotype, when either the CTG tract length, RNA expression levels, stability or context are less than optimal.

A noteworthy exception to this rule is the production of a DM1 phenocopy that results from the inducible expression of GFP sequences linked to the normal DMPK 3′UTR in mice [43]. No foci are observed in this study as the transgene encodes only 5 CTG repeats. Surprisingly, both severe cardiac and skeletal muscle pathology in conjunction with RNA splice defects are observed in these animals [43]. However, both our current study and those of others, demonstrate that expression of similar transgenes, in which GFP sequences are linked to the normal DMPK 3′UTR, in myoblast cell cultures is insufficient to cause aberrant RNA splicing or dysregulate myoblast differentiation [Figure 7, 44]. Thus the precise mechanism that underlies both the heart and skeletal muscle phenotypes observed in this phenocopy has yet to be completely understood. As DM1 patients are characterized by expanded CTG tracts, it is likely that the mechanistic basis for the pathology observed in this mouse strain differs in important ways from that observed in DM1 patients.

As noted above, a study by Wang and colleagues demonstrates that inducible expression of 960 interrupted CUG repeats located in the DMPK 3′UTR sequence results in the development of nuclear foci concurrent with arrhythmias, cardiomyopathy, cystolic and diastolic dysfunction and aberrant splicing [24]. In this study, we examined cardiac specific expression of 400 uninterrupted CUG repeats located in the 3′ of the β-galactosidase gene. In contrast to the results obtained by Wang et al., LacZ-(CUG)_400_ RNAs aggregate exclusively in the cytoplasm and do not result in aberrant splice site selection in several RNAs implicated in DM1 including *Tnnt2*, *Alp*, *Zasp* and *m-Titin* [Figure 6]. Consistent with the lack of splicing defects mild cardiac dysfunction was observed in α-MHC-LacZ-(CTG)_400_ mice. Specifically, structural analysis did not demonstrate gross abnormalities or hypertrophy. Ultra structural defects in mitochondria were observed in some sections [Figure 9], however such abnormalities were not widespread in the α-MHC-LacZ-(CTG)_400_ mice. The molecular defects that contribute to the observed mitochondrial abnormalities are unclear, but may be the result of modest decreases in Mbnl1 levels or function in the cytoplasm or alternatively are due to the increased steady-state Cug-bp1 levels. ECG data obtained in conscious α-MHC-LacZ-(CTG)_400_ mice was normal, however sedated α-MHC-LacZ-(CTG)_400_ TG*^low^* animals showed increased incidence of intraventricular conduction delay or bundle branch block and a significantly longer QRS duration [Tables 7 & 8]. Thus, the infra-Hisian conduction defect in α-MHC-LacZ-(CTG)_400_ mice may be subtle and may become unmasked with concomitant medications which slow conduction, such as anaesthetics. It is currently unclear why the α-MHC-LacZ-(CTG)_400_TG*^low^* mice demonstrate a more severe phenotype than the α-MHC-LacZ(CTG)_400_TG*^high^* mice. These differences may reflect the relatively subtle nature of the pathology and large numbers of mice in both groups may need to be analyzed to accurately assess differences in phenotypes that result from varying levels of transgene expression. These data therefore demonstrate that cytoplasmic LacZ-CUG foci are relatively benign and are not sufficient to either dysregulate splicing of a set of RNAs currently implicated in DM1 or result in significant cardiac pathology *in vivo*.

As the α-MHC promoter contains two exons and two intervening introns [28] the lack of transgene RNA splicing is unlikely to be the reason for the absence of nuclear CUG aggregation in α-MHC-LacZ-(CTG)_400_ mice. Intrinsic defects in the CTG tracts are also unlikely to be responsible for the lack of toxicity of LacZ-(CUG)_400_ RNAs, as these repeat sequences demonstrate marked instability that characterizes uninterrupted repeat tracts [29], [30]. Low steady-state levels of the LacZ-(CUG)_400_ RNAs could be a feature that influences the relatively benign phenotype. However, comparable expression levels of LacZ-(CUG)_400_ RNAs to that achieved by a DMPK minigene cassette encoding expanded CTG repeats was not sufficient to dysregulate splice site choice in human myoblasts cultures [Figure 8]. Therefore, the lack of toxicity of the LacZ-(CUG)_400_ RNAs could be either due to the cytoplasmic location of the CUG aggregates or alternatively to the lack of key regulatory elements in sequences that flank the repeat. Our data cannot distinguish between these two possibilities. In this regard it is important to note a study by Mastroyiannopoulos and colleagues, which demonstrates that expanded CUG tracts when expressed in the context of the DMPK 3′UTR, aggregate within the nucleus in myoblast cultures and prevent normal differentiation. Significantly, when this transgene is engineered to encode the woodchuck post transcriptional element that results in forced localization of the mutant RNA to the cytoplasm, the toxicity associated with these RNAs disappears, and they are no longer capable of dysregulating normal myoblast differentiation [27]. Thus these studies when taken together support the hypothesis that localization of CUG repeat RNA within the cytoplasm is an important variable, which is sufficient to render such RNAs non-toxic both in cell culture experiments and in the context of the whole animal.

Expression of CUG tracts results in aberrant RNA splicing due to its effect on a set of alternative splice factors, which include MBNL1 and CUG-BP1 [25], [26], [32], [33], [39], [45]–[49]. Specifically, expression of expanded CUG repeat sequences results in elevated steady-state CUG-BP1 protein levels, which plays a causative role in dysregulating splice site choice in a set of physiologically important RNAs implicated in DM1 both in cell culture and transgenic mouse experiments [25], [33], [39], [45]–[49]. However, our previous results in DM1 myoblasts demonstrate that siRNA mediated reduction of CUG-BP1 levels does not correct aberrant IR splicing [46], [47]. Thus these results demonstrate that although increased steady state levels of CUG-BP1 is sufficient to produce features of DM1, elevated CUG-BP1 levels may not be required for DM1 pathology to manifest, as reduction of CUG-BP1 levels does not correct the splice defects in DM1 myoblasts.

Inactivation of MBNL1 plays an important role in etiology of DM1 spliceopathy. Specifically, disruption of *Mbnl1* in mice is sufficient to recapitulate key features of the disease [32]. We have shown that re-expression of MBNL1 is sufficient to rescue the IR splice defects in DM1 patient cells [46], [47]. Consistent with our results, over expression of MBNL1 in transgenic mice expressing CTG tracts allows the rescue of both the splice defects and myotonia [50]. These data therefore demonstrate first, that functional inactivation of MBNL1 is sufficient to produce DM1 pathophysiology. Second, as MBNL1 mediated rescue serves to correct key features of DM1, inactivation of MBNL1 must be a necessary event that is required for the development of DM1 pathophysiology. The mechanism of MBNL1 inactivation is currently unknown. As previous studies have shown marked sequestration of MBNL1 in CUG foci both in skeletal muscle and heart cells (35,36), it has been hypothesized that MBNL1 depletion occurring as a consequence of aberrant sequestration, is a key mechanism that underlies the functional inactivation of MBNL1 in DM1.

As splice defects are not observed in α-MHC-LacZ-(CTG)_400_ mice, we studied the ability of the LacZ-(CUG)_400_ RNA to alter the stoichiometry or behavior of Mbnl1 and Cug-bp1. Similar, but relatively small amounts of endogenous MBNL1, localize both in nuclear CUG foci (∼8%) in DM1 myoblasts, where aberrant splicing is observed, and in cytoplasmic CUG-foci (∼7%) in α-MHC-LacZ-(CTG)_400_ cardiomyocytes, where splice defects are not observed [Figure 4]. As experiments in *Mbnl1* ^+/−^ mice demonstrate that an ∼50% decrease in Mbnl1 is insufficient to alter splice site choice [32], these data demonstrate that aggregation per se cannot be the sole mechanism that underlies MBNL1 inactivation in DM1 cells. Nonetheless, MBNL1 inactivation must be a key event in DM1 myoblasts as over expression of MBNL1 is sufficient to rescue the splice defects in these cells [46], [47]. Thus as expression of LacZ-(CUG)_400_ RNAs is not sufficient to dysregulate RNA splicing, these RNAs must also by inference be unable to inactivate Mbnl1 function *in vivo*. The molecular basis of MBNL1 inactivation in DM1 myoblasts is currently unclear and is an important area of future investigation.

Significantly, steady state Cug-bp1 levels are elevated ∼2.4 and ∼1.5 fold in α-MHC-LacZ- (CTG)_400_TG*^high^* and α-MHC-LacZ-(CTG)_400_TG*^low^* mice respectively, at six months of age [Figure 5]. Experiments carried out by Lin and colleagues demonstrate that expression of ∼250 CTG repeats located in the 3′UTR of α-actin gene does not result in elevated Cug-bp1 levels in mouse skeletal muscles [35]. These data demonstrate that CTG tracts, greater than 250 repeats, may be necessary to increase steady state Cug-bp1 levels, in adult mouse tissues. On inducible expression of very high levels of interrupted CTG tracts, Cug-bp1 levels are increased in the heart [24]. However as Cug-bp1 levels were not quantitated in this study, the exact increases in Cug-bp1 levels at time points at which splice defects manifest are unclear. Cug-bp1 over expression studies in mice demonstrate that a 4–6 fold elevation in Cug-bp1 levels [48] may be required to result in aberrant splicing. Therefore, in adult mouse tissues very high levels of Cug-bp1 may be necessary to elicit splice defects. If adult human tissues behave in a similar fashion, CUG-BP1 levels, which are high enough to dysregulate splicing, may result only in conjunction with the expression of very large CTG tracts. In this event, elevated CUG-BP1 levels may be more relevant in the etiology of congenital DM1 rather than adult onset DM1.

Our results show that CTG tracts are necessary but not sufficient to cause nuclear aggregation of CUG repeats. The precise RNA motifs required for nuclear aggregation of CUG repeats are currently unknown; however, the presence of such motifs must play a key role both in determining the ultimate location of the CUG repeat encoding RNAs. In this respect it is significant to note that both MBNL1 and hnRNP H have been shown to be required for nuclear focus formation [46], [51]. Specifically, we have shown that siRNA mediated inactivation of MBNL1, which binds to the stem of the CUG hairpin, results in increased dispersion of nuclear foci in DM1 cells [46]. siRNA mediated reduction of hnRNP H, which binds to the base of the CUG hairpin, has also been demonstrated to allow transport of RNAs encoding expanded CUG repeats into the cytoplasm [51]. These results suggest that proteins that either bind to CUG hairpins or to key regions of the flanking sequence may facilitate hairpin stabilization and aggregate formation in the nucleus. As aggregation and transport into the cytoplasm may be two competing events within the nucleus, these data predict that small molecules, which serve to decrease the rate of aggregate formation in the nucleus, to allow transport of the toxic CUG RNAs into the cytoplasm, may be sufficient to greatly ameliorate DM1 symptoms in patients whose repeat tract sizes are not long enough to elicit large increases in CUG-BP1 levels. Thus small molecules that decrease the rate of either MBNL1 or hnRNP H binding to the mutant *DMPK* RNA may serve to increase its transport out of the nucleus. Such a cadre of drugs is attractive as they may prove to be less toxic, when compared to small molecules that abolish the interaction of these proteins with the mutant *DMPK* RNA, as their disruptive effect on MBNL1 or hnRNP H interactions with their normal target RNAs may be less severe.

## Materials and Methods

### Cell culture and transfection

Normal and DM1 myoblasts were a generous gift from Dr. Charles Thornton. DM1 fibroblasts were purchased from Coriell Institute for Medical Research, NJ, U.S.A. Normal and DM1 myoblast and fibroblasts cultures were immortalized by infection with the SV40 virus. Myoblast cultures and lines were maintained in SKGM medium (Lonza Inc., USA) containing 10% fetal bovine serum and fibroblast cultures and lines were maintained in MEM medium containing 20% fetal bovine serum.

### FISH analyses

*In situ* fluorescence hybridization (FISH) analyses were carried out primarily as described by Taneja et al, 1995 [15] and Dansithong et al, 2005 [46]. Briefly, cardiomyocytes were prepared from mouse hearts using standard protocols [52] and plated immediately on chamber slides and fixed in 4% paraformaldehyde in PBS for 20 minutes at room temperature and stored in 70% ethanol at 4°C. Endogenous MBNL1 was detected using MBNL1 monoclonal antibody (MB1a) at a dilution of 1∶200 [31]. Secondary antibody, anti-mouse IgG conjugated with FITC (Alexa Fluor 488, Molecular probes, USA) was used at a dilution 1∶2000. For FISH studies, a Cy3 conjugated (CAG)_10_ oligonucleotide probe (Operon, USA) was used to detect CUG repeat expansions as described by Taneja et al, 1995 [15]. To estimate the amount of MBNL1 present in the nucleus, the cytoplasm and the foci, the fluorescence signals (area×intensity) were measured using IP Lab software (Scanalytics Inc., USA). The percentage of MBNL1 present in the nucleus and cytoplasm of cardiomyocytes derived from α-MHC-LacZ mice and normal human myoblasts was calculated as: MBNL1^nucleus^ = [MBNL1^nucleus^/MBNL1^whole cell^]×100; MBNL1^cytoplasm^ = [(MBNL1^whole cell^−tMBNL1^nucleus^)/MBNL1^whole cell^]×100. For cardiomyocytes derived from α-MHC-LacZ-(CTG)_400_ mice the percentage of MBNL1 in nucleus, cytoplasm and foci was calculated as: MBNL1^nucleus^ = [MBNL1^nucleus^/MBNL1^whole cell^]×100; MBNL1^cytoplasm^ = [(MBNL1^whole cell^t−(MBNL1^nucleus^+MBNL1^cytoplasmic foci^)/MBNL1^whole cell^]×100 ; MBNL1 ^cytoplasmic foci^ = MBNL1^cytoplasmic foci^/MBNL1^whole cell^×100. In DM1 myoblasts the percentage of MBNL1 in nucleus, cytoplasm and foci was calculated as: MBNL1^nucleus^ = [MBNL1^nucleus^−MBNL1^nuclear foci^/MBNL1^whole cell^]×100; MBNL1^cytoplasm^ = [(MBNL1^whole cell^−(MBNL1^nucleus^+MBNL1 ^nuclear and cytoplasmic foci^)/MBNL1^whole cell^]×100; MBNL1^nuclear and cytoplasmic foci^ = MBNL1^nuclear and cytoplasmic foci^/MBNL1^whole cell^×100. FISH was performed on frozen sections (6 µm) using (CAG)_10_-Cy3 probes as described by Mankodi et al, 2000 [37]. After hybridization, the sections were mounted with mounting medium (Vector, USA). In all cases the images were examined using a LSM 510 Confocal microscopy in the Doheny Eye Institute at the University of Southern California.

### Generation of α-MHC-LacZ-(CTG)_400_ and α-MHC-LacZ mice

(CTG)_400_ tracts were cloned into an *Sfi*I site located within a linker sequence (TGGCCACGCGGGCCATTTAAATGGCCATTAGGGCC : *Sfi*I sites are underlined) that was inserted between the termination codon of the β-galactosidase gene and the bovine growth hormone polyadenylation (BGH-PolyA) sequence. The α-myosin heavy chain (α-MHC) promoter was used to drive expression of the transgene [28]. Both the α-MHC-LacZ-(CTG)_400_ cassette and control α-MHC-LacZ cassette, which did not encode an expanded CTG tract were injected into fertilized C57BL/6J mouse eggs to generate two α-MHC-LacZ-(CTG)_400_ lines and two control α-MHC-LacZ lines [α-MHC-LacZ-(CTG)_0_]. The transgenic mice were genotyped by PCR using β-galactosidase specific primers (forward 5′-ATGATAGATCCCGTCGTTTT ACAAC-3′ and reverse 5′-TCAATCAGCGTGCCGTCG GCGGTG-3′).

### Southern and Northern blot analyses

For Southern blot analyses, genomic DNA from transgenic mice tails was digested with *Pvu*II and for Northern blot analyses, total RNA from transgenic heart tissue was isolated using Trizole (Invitrogen, USA) according to the manufacturer's protocol. The blots were hybridized with radioactive probes using standard techniques. The probe used for Southern blot analyses consists of a 280 bp fragment of the BGH-PolyA sequence shown in Figure 2; Panel A. β-galactosidase (bases 300–815) and *Gapdh* specific probes were used to detect transcripts in Northern blot analyses.

### Western blot analyses

Heart tissue was collected from transgenic mice and extracts were prepared by homogenization with the extraction buffer [25 mM Tris-HCl pH 7.6, 400 mM NaCl, 0.5% NP40, 5 mM EDTA, 25% Sucrose, 2 mM PMSF and Protease inhibitor (Sigma Inc., Catalog # P-8340)]. Tissue extracts were incubated on ice for 1 hour and then centrifuged for 20 min at 20,000×g. Equal amounts of protein (either 6 µg or 10 µg) were resolved by SDS-PAGE and transferred to Hybond P membranes (Amersham Bioscience Inc., USA). After blocking with 5% skim milk in 0.1% Tween 20 in PBS, the membranes were incubated with primary antibodies for 2 hrs at room temperature or overnight at 4°C. The membranes were washed with 0.1% Tween 20 in PBS and subsequently incubated with the corresponding secondary antibodies conjugated with HRP. Signals were detected by using the ECL plus detection reagents (Amersham Bioscience Inc., USA) according to the manufacturer's protocol. The primary antibodies were CUG-BP1 [3B1 monoclonal antibody (200 µg/ml), Santa Cruz Inc. (catalog # sc-20003), 1∶4000], and MBNL1 monoclonal antibody (MB1a, 1∶3000) [31] to detect the Cug-bp1 and Mbnl1. To control for protein quality and loading the membranes were re-probed with goat anti-GAPDH [V-18 polyclonal antibodies (200 µg/ml), Santa Cruz Inc. (catalog # sc-20357)] at a dilution of 1∶3000. The secondary antibody dilutions were 1∶8000 for goat anti-mouse IgG-HRP [(1 mg/ml), Sigma Inc. (catalog # A2304)], and 1∶5000 for donkey anti-goat IgG-HRP [(400 µg/ml), Santa Cruz Inc. (catalog # sc-2056)]. The relative band intensities were measured by densitometry analyses using Gene Tool (Syngene Inc., USA). To ensure that the signals were not saturated, prior standardization experiments were carried out as described in Paul et al, 2006 [47].

Sub-cellular fractions (cytoplasmic and nuclear) from mouse heart tissue was prepared using methods primarily described by Charlet et al, 2002 [49] with some modifications. Heart tissues were collected from transgenic mice and homogenized with cold lysis buffer (25 mM Tris–HCl [pH 7.6], 10 mM NaCl, 1.0 mM EDTA, 2.0 mM MgCl_2_, 0.5 mM DTT, and protease inhibitor). The soluble fraction was passed through a 26-gauge needle eight times using a 1 ml syringe. Nuclear: cytoplasmic separations were carried out by centrifugation twice at 600×g for 5 min. The cytoplasmic extract was prepared by centrifuging the supernatant fraction for 20 min at 20,000×g. The pellet from the nuclear: cytoplasmic separations was suspended in nuclear extraction buffer (25 mM Tris–HCl [pH 7.6], 600 mM NaCl, 0.05% NP40, 1.0 mM EDTA, 2.0 mM MgCl_2_, 0.5 mM DTT, and protease inhibitor) and centrifuged for 20 min at 20,000×g. The resulting supernatant was denoted as a nuclear extract. Equal amounts of protein (10 µg) from the nuclear and cytoplasmic fractions were resolved by SDS-PAGE and transferred to Hybond P membranes. The membranes were probed with TBP (TATA binding protein) monoclonal antibody [(1.99 mg/ml), Abcam Inc. (catalog # ab818), 1∶5000], which was used as a nuclear marker and goat anti-GAPDH [V-18 polyclonal antibodies (200 µg/ml), Santa Cruz Inc. (catalog # sc-20357), 1∶3000], which was used as a cytoplasmic marker. The secondary antibody dilutions were 1∶8000 for goat anti-mouse IgG-HRP [(1 mg/ml), Sigma Inc. (catalog # A2304)], and 1∶5000 for donkey anti-goat IgG-HRP [(400 µg/ml), Santa Cruz Inc. (catalog # sc-2056)].

### RT-PCR analyses of RNA isolated from transgenic mice

Total RNA was isolated from transgenic mouse hearts using Trizole (Invitrogen, USA) according to the manufacturer's protocol. cDNA was synthesized from 5 µg of total RNA using the cDNA synthesis kit (Amersham Bioscience Inc., USA) and PCR was carried out using 150 ng of the synthesized cDNA. PCR amplification was carried out for 25 and 30 cycles for each gene. The sequences of the primers used for the amplification of *Tnnt2*, *Alp*, *Zasp*, and *m-Ttn* are: *Tnnt2* (forward 5′-GCCGAGGAGGTGGTGGAGGAGTA-3′, reverse 5′-G TCTCAGCCTCACCCTCAG GCTCA-3′), *Alp* (forward 5′-AGCTGCCAAtCCTGTGTCCTG-3′, reverse 5′-GATCCTGCAGCACCCTGAAG-3′), *Zasp* (forward 5′-GGAAGATGAGGCTGATGAGTGG-3′, reverse 5′-TGCTGACAGTGGTAGTGCT CTTTC-3′), and *m-Ttn* (forward 5′-GTGTGAGTCGCTCCAGAAACG-3′ reverse 5′-CCACCACAGG ACCATGTTATTTC-3′). To ensure that the signals were not saturated, prior standardization experiments were carried out as described in Paul et al, 2006 [47]. The PCR products were cloned and verified by sequencing. The relative band intensities were measured by densitometry analyses using Gene Tool. Percent of exon inclusion was calculated as [exon inclusion/(exon inclusion+exon exclusion)]×100.

### Construction of CTG expression plasmids

DMPK 11-15(CTG)_5 or 300_ plasmids encoding *DMPK* exons 11-15 (2.3 kb), containing either 5 CTG repeats or 300 interrupted CTG repeats in exon 15, was cloned into pAdTrac-CMV (ATCC, USA) using techniques described by Philips et al, 1998 [33]. The interrupted repeats are composed of repeating units of the sequence (CTG)_20_CTCGA. The GFP-DMPK 3′UTR(CTG)_5_ plasmid was constructed by cloning a 0.7 kb sequence located immediately 3′ of the human DMPK termination codon, 3′ of the GFP sequences in pEGFP-C1 (BD Clontech, USA). GFP-DMPK 3′UTR(CTG)_400_ was constructed by cloning an uninterrupted tract of ∼400 CTG repeats at the site of the existing (CTG)_5_ repeat sequence in the GFP-DMPK 3′UTR(CTG)_5_ plasmid. The LacZ-(CTG)_0 or 400_ plasmids were constructed by cloning cassettes encoding the β-galactosidase gene followed by a tract of either 0 or ∼400 uninterrupted CTG repeats and the BGH-PolyA sequence at 3′ of the CMV promoter in pcDNA3.1 [Invitrogen, USA]. The integrity of the CTG repeats was confirmed by restriction enzyme analysis and sequencing.

### RT-PCR and Real-time PCR analyses of RNA isolated from human myoblasts

Normal myoblasts were transfected with 6 different cassettes under the transcriptional control of the CMV promoter as described previously [46]. Briefly, the transfected cells were selected by treating with G418 (300 µg/ml) for 5 days. Total RNA was isolated using the RNAeasy mini kit (Qiagen Inc., USA) according to the manufacturer's protocol. DNAse I treated RNAs were used to synthesize cDNA using MMLV reverse transcriptase and random hexanucleotide primers. PCR analyses were carried out as described above. The sequences of the primers used for the amplification of insulin receptor (*IR*), cardiac troponin T (*cTNT*) and *GAPDH* RNAs are: *IR* (forward 5′-CCAAAGACAGACTCTCAGAT-3′, reverse 5′-AACATCGCCAAGGGACCTGC-3′), *cTNT* (forward 5′-ATAGAAGAGGTGGTGGAAGA GTAC-3′, reverse 5′-GTCTCAGCCTCTGCTTCAGCATCC-3′), *GAPDH* (forward 5′-TGAAGGTCG GAGTCAACGGATTTGG-3′, reverse 5′-GGAGGCCATGTGGGCCATGAG-3′). To assess the steady-state expression levels of the β-galactosidase gene and DMPK minigene encoding expanded CTG tracts, synthesized cDNA (100 ng) from normal myoblasts expressing LacZ-(CTG)_0 or 400_ and DMPK 11-15(CTG)_5 or 300_ were subjected to RT-PCR analysis. The primers sequences used for the amplification of LacZ-(CTG)_0 or 400_ and DMPK 11-15(CTG)_5 or 300_ are: LacZ-(CTG) _0 or 400_ constructs (forward 5′-ATGAT AGATCCCGTCGTTTTACAAC-3′ and reverse 5′-TCAATCAGCGTGCCGTCGGCGGTG-3′) and DMPK 11-15(CTG)_5 or 300_ constructs (forward 5′-GAAGGCAGCAAGCCGGGCCGTCCG-3′, reverse 5′-AGGGGGAGGTGTGGGAGGTTTTTT-3′). *GAPDH* was amplified in parallel as an internal control for RNA quality and the reverse transcriptase reaction. The relative band intensities were measured by densitometry analyses using Gene Tool. To ensure that the signals were not saturated, prior standardization experiments were carried out as described in Paul et al, 2006 [47]. Signals were normalized to the expression of GAPDH and tabulated as percent relative expression. Real-time PCR was carried out to quantitate the expression levels of LacZ-(CUG)_400_ and DMPK 11-15(CUG)_300_ transcripts. The primers used in Real-time PCR are: LacZ-(CTG)_400_ constructs (forward 5′-ATGATAG ATCCCGTCGTTTTAC-3′, reverse 5′-CGCCATTCAGGCTGCGCAACTGTTG-3′), DMPK 11-15(CTG)_300_ constructs (forward 5′-GAATGCACTGAGACCCCGACATTCC-3′, reverse 5′-ATTATGA TCAGTTATCTAGATCCGG-3′), and GAPDH (forward 5′-CCACCCATGGCAAATTCCATG-3′, reverse 5′-TGATGGGATTTCCATTGATGAC-3′). The PCR reaction mixture contained SYBR Green PCR Master Mix (Bio Rad Inc., USA) and 0.5 pmol primers. PCR amplification was carried out for 45 cycles: denaturation at 95°C for 30 sec, annealing at 60°C for 30 sec and extension at 72°C for 1 min.

### Electrocardiography Studies

A total of 35 adult C57BL/6J strain mice were studied. The mean age was 27±8 weeks and mean weight was 30±5 grams of all mice. Mice were anesthetized with intraperitoneal pentobarbital (0.033 mg/gm each). A 6-lead surface ECG was obtained with 25 gauge electrodes placed subcutaneously in each limb. Mean sinus-cycle length (SCL), heart rate (HR), PR, QRS, and QT intervals were measured for each animal as described by Berul et al, 1996 [53]. A rate corrected QT interval (QTc) was calculated using a murine formula [54].

### Ambulatory Electrocardiogram Telemetry

Radiofrequency transmitters (DataSciences International, St. Paul, MN, USA) were implanted into a subcutaneous pocket with leads secured under the upper right and left limbs to record lead I ECG. After a 48-hours recovery period, ECGs were recorded continuously for 10 minutes. All baseline telemetry measurements for SCL, PR, QRS and QT intervals were made for three consecutive cardiac cycles by an experienced observer blinded to the genotypes of the mice.

### Exercise Tolerance Test

Animals with implanted transmitters were exercised on a multilane graded treadmill machine (Exer-6M, Columbus Instruments, Columbus, OH, USA). Mice were encouraged to run for 30 minutes at a constant speed of 12.5 m/min at a slope of 15 degrees. Telemetry ECG recordings and measurements were made at rest, after 10 and 20 minutes of exercise, just before termination of exercise, and at recovery. During exercise and recovery, telemetric ECG recordings were examined for conduction abnormalities and the presence of inducible arrhythmias.

### Statistical Analysis

Statistical analyses were performed with SPSS software version 11.5 for Windows. Continuous variables, such as ECG intervals, cardiac conduction properties and echocardiography measurements were compared for genotypes using an analysis of variances (ANOVA) test followed by post-hoc analysis. Pearson chi-square tests were performed for categorical data. Values are presented as the mean±1 standard error of mean (SEM). The Student t-test was used to analyze data derived from RNA splicing, expression analyses and the examination of MBNL1 cellular localization. Statistical significance was established with a *p* value of <0.05.

## Supporting Information

Figure S1 Images of DM1 cells containing both nuclear and cytoplasmic foci. Panels A–E: Nuclear DAPI stains of normal and DM1 myoblast and fibroblast cultures and SV40 transformed lines are shown in the upper set of panels. Mutant DMPK transcripts encoding the expanded CUG tracts are detected by hybridization with a (CAG)10-Cy3 probe (middle panels). The lower set of panels show merged images of DAPI and (CAG)10-Cy3 stains, demonstrating the nuclear and cytoplasmic location of the CUG RNA foci in these cells. Transcripts containing expanded CUG repeats are not observed in the normal myoblasts and fibroblasts. In DM1 cells, ∼70% and ∼30% of all sampled cells [number of cells counted in each case are shown in Figure 1; Table 1 of the main text] showed nuclear foci or both nuclear and cytoplasmic foci, respectively.
(2.66 MB TIF)

Figure S2 MBNL1 monoclonal antibody (MB1a) specifically detects Mbnl1 in mouse cardiomyocytes. Nuclear DAPI stains of cardiomyocytes derived from wild type, and Mbnl1−/− mice (a gift from Dr. Swanson MS) are shown in a and c. Distribution of endogenous Mbnl1 is visualized as a green signal in wild type cardiomyocytes (b) using anti-MBNL1 (MB1a) monoclonal antibody and a secondary antibody (anti-mouse IgG) conjugated with FITC. Mbnl1 is not detected in Mbnl1−/− cardiomyocytes (d).
(3.93 MB TIF)
