# Supplementary figures and images for: Cytoplasmic CUG RNA Foci Are Insufficient to Elicit Key DM1 Features
Source: PLoS One. 2008 Dec 18;3(12):e3968. doi: 10.1371/journal.pone.0003968 (PMC2597774; doi:10.1371/journal.pone.0003968)

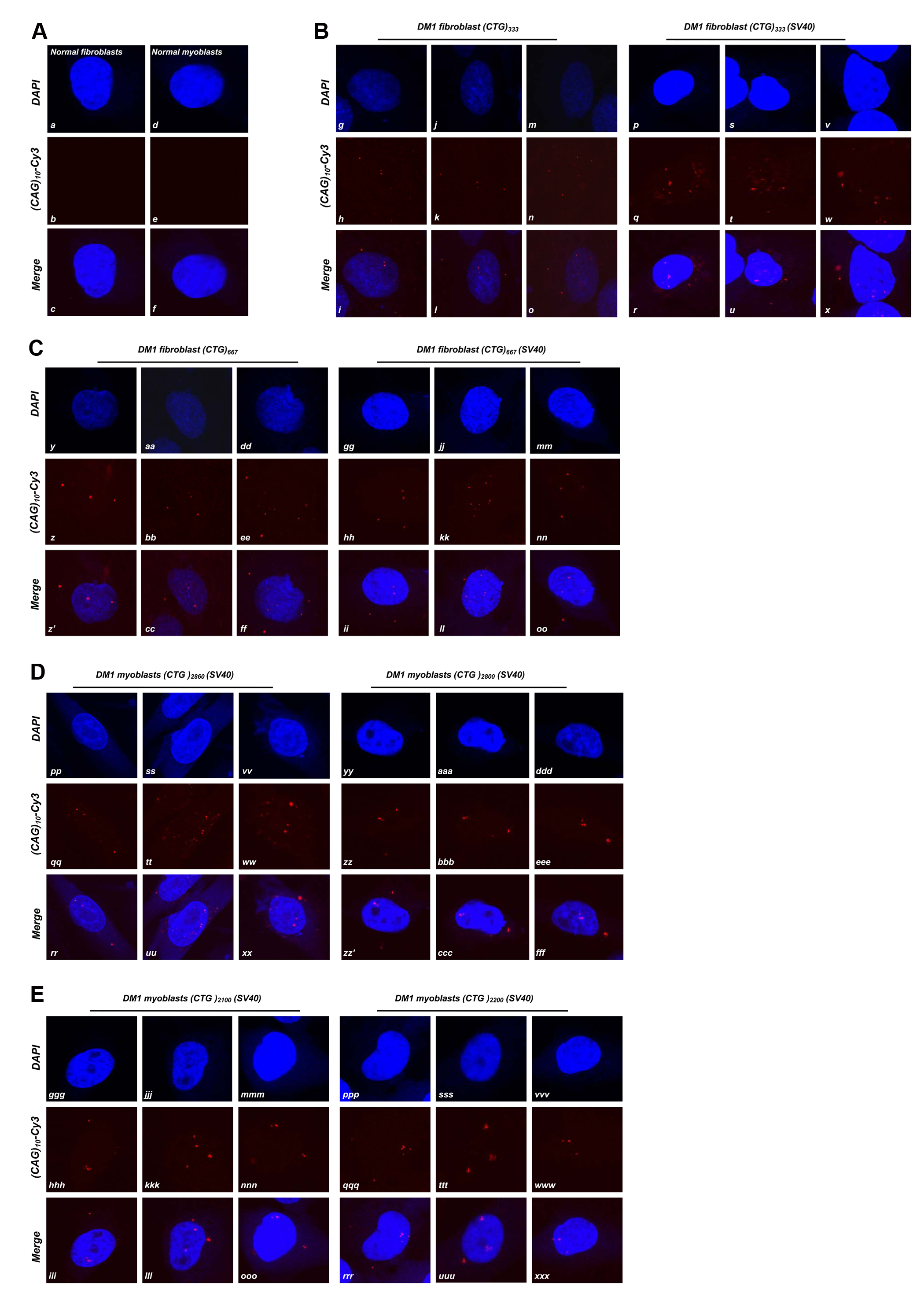

Supplement: Figure S1 — Images of DM1 cells containing both nuclear and cytoplasmic foci. Panels A–E: Nuclear DAPI stains of normal and DM1 myoblast and fibroblast cultures and SV40 transformed lines are shown in the upper set of panels. Mutant DMPK transcripts encoding the expanded CUG tracts are detected by hybridization with a (CAG)10-Cy3 probe (middle panels). The lower set of panels show merged images of DAPI and (CAG)10-Cy3 stains, demonstrating the nuclear and cytoplasmic location of the CUG RNA foci in these cells. Transcripts containing expanded CUG repeats are not observed in the normal myoblasts and fibroblasts. In DM1 cells, ∼70% and ∼30% of all sampled cells [number of cells counted in each case are shown in Figure 1; Table 1 of the main text] showed nuclear foci or both nuclear and cytoplasmic foci, respectively. (2.66 MB TIF) [file pone.0003968.s001.tif]

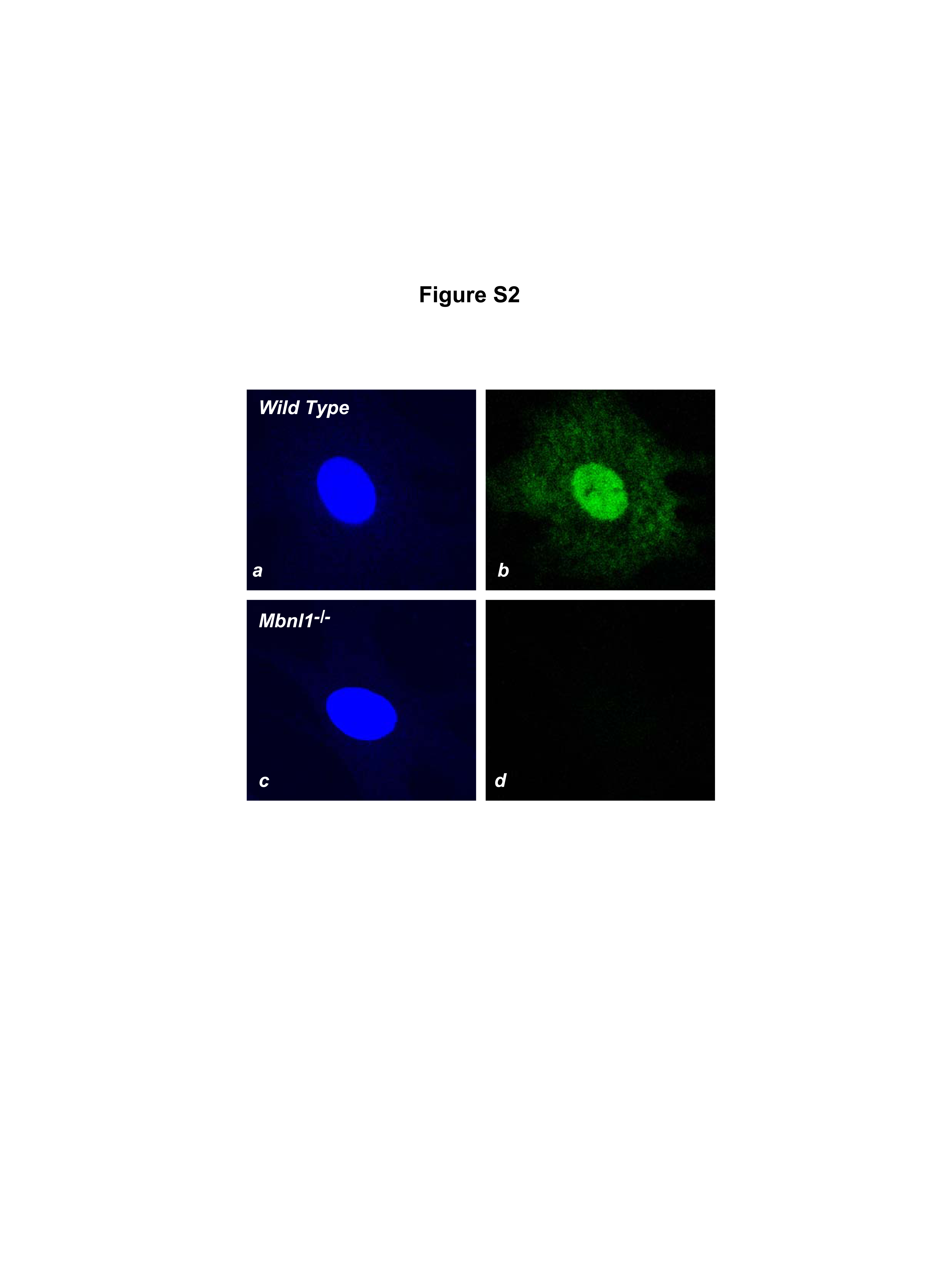

Supplement: Figure S2 — MBNL1 monoclonal antibody (MB1a) specifically detects Mbnl1 in mouse cardiomyocytes. Nuclear DAPI stains of cardiomyocytes derived from wild type, and Mbnl1−/− mice (a gift from Dr. Swanson MS) are shown in a and c. Distribution of endogenous Mbnl1 is visualized as a green signal in wild type cardiomyocytes (b) using anti-MBNL1 (MB1a) monoclonal antibody and a secondary antibody (anti-mouse IgG) conjugated with FITC. Mbnl1 is not detected in Mbnl1−/− cardiomyocytes (d). (3.93 MB TIF) [file pone.0003968.s002.tif]
